# High-resolution tracking of unconfined zebrafish behavior reveals stimulatory and anxiolytic effects of psilocybin

**DOI:** 10.1038/s41380-023-02391-7

**Published:** 2024-01-17

**Authors:** Dotan Braun, Ayelet M. Rosenberg, Elad Rabaniam, Ravid Haruvi, Dorel Malamud, Rani Barbara, Tomer Aiznkot, Berta Levavi-Sivan, Takashi Kawashima

**Affiliations:** 1 Department of Brain Sciences, Weizmann Institute of Science, 234 Herzl Street, Rehovot, Israel; 2 The Jerusalem Mental Health Center, Jerusalem, Israel; 3 Department of Animal Sciences, The Robert H. Smith Faculty of Agriculture, Food, and Environment, Hebrew University of Jerusalem, 229 Herzl Street, Rehovot, Israel

**Keywords:** Neuroscience, Physiology, Drug discovery

## Abstract

Serotonergic psychedelics are emerging therapeutics for psychiatric disorders, yet their underlying mechanisms of action in the brain remain largely elusive. Here, we developed a wide-field behavioral tracking system for larval zebrafish and investigated the effects of psilocybin, a psychedelic serotonin receptor agonist. Machine learning analyses of precise body kinematics identified latent behavioral states reflecting spontaneous exploration, visually-driven rapid swimming, and irregular swim patterns following stress exposure. Using this method, we found that acute psilocybin treatment has two behavioral effects: [i] facilitation of spontaneous exploration (“stimulatory”) and [ii] prevention of irregular swim patterns following stress exposure (“anxiolytic”). These effects differed from the effect of acute SSRI treatment and were rather similar to the effect of ketamine treatment. Neural activity imaging in the dorsal raphe nucleus suggested that psilocybin inhibits serotonergic neurons by activating local GABAergic neurons, consistent with psychedelic-induced suppression of serotonergic neurons in mammals. These findings pave the way for using larval zebrafish to elucidate neural mechanisms underlying the behavioral effects of serotonergic psychedelics.

## Introduction

Mood-related mental disorders cast significant socioeconomic impacts on modern societies, and 5% of adults are estimated to suffer from depression globally [1]. The serotonin theory of depression [2] emerged soon after the discovery of the serotonergic system in the brain in the 1960s [3]. Since then, the serotonergic system has been a therapeutic target for major depression, obsessive-compulsive disorders, and other psychiatric disorders [4]. Current medication regimens based on serotonin-selective reuptake inhibitors (SSRIs) have limited efficacies in terms of quickness of therapeutic effects [5] and final remission rates [6], calling for a better understanding of neural mechanisms for mood-related behavioral alternation and its pharmaceutical rescue.

The recent resurgence of the use of hallucinogenic drugs as fast-acting antidepressants has opened new opportunities for the research of neural circuit mechanisms critical for the treatment of mood-related disorders [7]. Psilocybin, a psychedelic compound, originates in the genus of gilled mushrooms *Psilocybe* and acts as a potent agonist for a family of serotonin receptors. Psilocybin is effective for clinical cases of treatment-resistant depression [8], and only a few doses can have lasting effects on depression symptoms for months or even up to a year [9, 10]. These reported therapeutic effects are markedly different from the short-lasting effects of other classes of psychedelics such as ketamine [11], making psilocybin and its derivatives a promising class of drugs for treating mood-related disorders.

We have a limited understanding of how psilocybin exerts its therapeutic effects in the brain [12]. Human brain imaging studies show that psilocybin alters the functional connectivity within the default mode network which includes the prefrontal cortex [13]. Microscopic observations of the prefrontal cortex in mice suggest that such changes might occur from the induction of new excitatory synapses [14]. There have also been efforts to derive HTR2 agonists that can prevent stress-induced behavioral changes without causing hallucination-like behaviors [15–17]. Very few studies have focused on changes in subcortical structures such as the midbrain, the brainstem and the cerebellum. One study showed that serotonergic psychedelics suppress the firing of serotonergic neurons in the dorsal raphe nucleus [18], of which behavioral significance is still a subject of active debate [19]. In general, the links between neural dynamics in subcortical structures and psilocybin’s behavioral effects have been challenging to investigate in mammals.

Larval zebrafish provide opportunities for studying the functions of brain structures that are not accessible in mammalian models. Its small size, optical transparency, and genetic accessibility allow optical recording of neural activity across the whole brain at a single-cell resolution [20–23]. It has a conserved raphe serotonergic system in the hindbrain/midbrain [24–31], in addition to teleost-specific serotonergic nuclei in the hypothalamus [24, 25, 32], that allows detailed investigation of the working principles of serotonergic neurons during behavior. Larval zebrafish have also been used to screen the behavioral effects of stress exposure, antidepressants, and genetic mutations [33–36]. However, few published studies have examined or discussed the possible behavioral effects of psychedelics in larval zebrafish [37–39], and, to date, there have been no reports on detailed behavioral effects of psilocybin. Such scarcity of behavioral insights impedes further research into their actions in neural circuit dynamics.

Tracking of millisecond-timescale body kinematics identified diverse types of latent behavioral states in larval zebrafish [40, 41], and these findings provided direct insights into underlying neural mechanisms [28, 42]. Yet, previous studies examining the effect of drug treatments on behavioral stress responses in zebrafish have generally focused on macroscopic parameters such as overall travel distance and environmental preference. The lack of body kinematics information in these studies makes it challenging to connect observed behavioral changes and underlying neural mechanisms. A barrier to using high-speed behavioral tracking to study stress responses is that the small chambers typically used for such behavioral tracking can themselves incur confinement stress [33]. Thus, precision approaches have yet to be explored for studying the effects of drug treatments on stress responses in zebrafish.

To overcome this challenge, we developed a machine-learning approach that tracks body kinematics in a large, unconfining environment and infers changes in behavioral states by stress exposure and psilocybin treatments. This approach enabled us to disambiguate distinct behavioral states governing spontaneous exploration, visually-driven rapid scooting, and irregular swim patterns after stress exposure. Acute psilocybin treatment facilitated rapid swimming in the absence of visual stimuli (stimulatory effect) and prevented occurrences of irregular swim patterns following stress exposure (anxiolytic effect). These effects on body kinematics were different from those of SSRIs and were rather similar to those of ketamine. Neural activity imaging experiments suggested that psilocybin suppresses the neural activity of serotonergic neurons in the dorsal raphe nucleus. These behavioral and neural effects parallel observations in mammals and humans and open new opportunities for studying how serotonergic psychedelics impact neural dynamics in evolutionarily conserved structures across vertebrates.

## Results

### High-resolution fish tracking system for a large environment

**Fig. 1:**
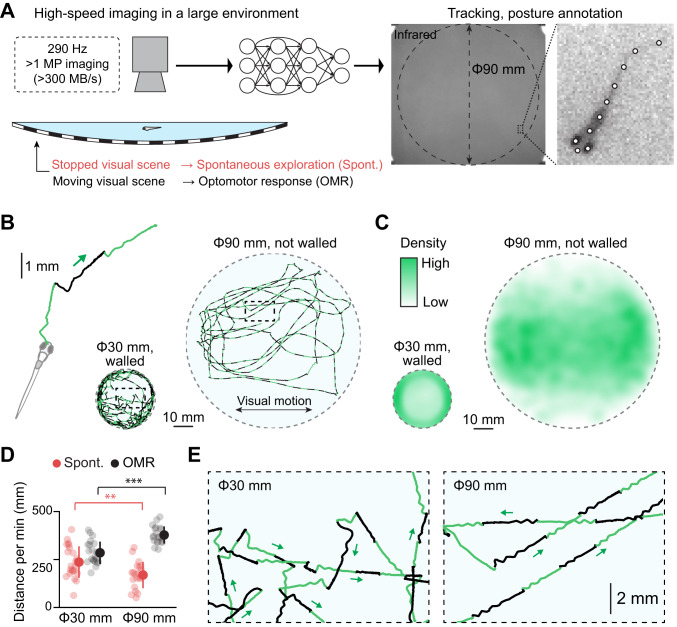
High-resolution, high-speed tracking of zebrafish behavior in a large environment. **A** Schematic illustration of our experimental setup and analysis pipeline. Fish swam in an arena where visual stimuli were projected underneath. We acquired high-resolution images at a speed of 290 Hz across the arena of up to 90 mm in diameter. Data analysis pipelines based on a deep neural network identified the fish loci and body postures. We measured spontaneous exploration while the visual stimuli are stopped (Spont.), and visually-induced optomotor response (OMR) while the visual stimuli are moving. See Fig. S1A and the “Methods” section for details. **B** Head centroid trajectories in a small, walled environment (30 mm) and a large, unwalled environment (90 mm). Each swim episode is colored in either light green or black to separate different swim episodes visually. **C** Distributions of head centroids during experiments across tested fish. *N* = 22 and *N* = 20 fish for the small and large dishes, respectively. **D** Swimming distance per minute during spontaneous exploration (red) and optomotor response (OMR, black). Transparent circles represent individual fish. **p = 0.0081; ***p = 1.7 × 10^−^^6^ from 2-sample *t*-test. Error bars represent standard deviations across tested fish. **E** Expanded head centroid trajectories from the outlined central parts of the small arena (left) and the large arena (right) from (**B**). The large environment facilitates straight swim patterns.

We developed an experimental setup and a data processing pipeline that examine how innate behaviors of larval zebrafish, such as spontaneous exploration and optomotor response, change after drug treatments and stress exposure. This setup tracks the precise body kinematics of a single fish in an environment that is large (90 mm) compared to the length of larval zebrafish (~4 mm) at a high-resolution (>1100 × 1100 pixels) and high-speed (290 Hz) (Fig. 1A, Fig. S1A). Fish behavior was recorded at infrared wavelengths for 15 min while visual gratings stopped for 10 s (spontaneous exploration) and moved for 10 s (optomotor response, OMR) in cycles (Fig. S1A, B). We tested a single fish per experiment to exclude the effect of social dynamics throughout this study. The large size of the imaged arena resulted in lower pixel resolution relative to previous body kinematics studies [40–42]. Therefore, our data processing tracked head trajectories and tail kinematics at subpixel resolution, i.e., at spatial scales smaller than the pixel size enabled by prior knowledge of the shape of the animal. We achieved localization accuracy at around 25 μm for the head trajectory (Fig. S1C).

Using this setup, we examined the behavioral impacts of the size of behavioral arenas by comparing swimming trajectories between those in a small, walled arena (30 mm) and those in our large, unwalled arena (90 mm). Zebrafish typically swam near the wall in the small arena due to their innate preference called thigmotaxis [43]. In our large arena, on the contrary, they explored widely (Fig. 1B, C) and swam longer distances during optomotor response (Fig. 1D). Spontaneous swimming distance was longer in the small arena, indicating an effect of confinement stress [33] (Fig. 1D). We also observed notable differences in swim trajectories around the central area of the dish. Fish showed frequent turning in the small arena even when they were not near the wall, potentially due to confinement stress, whereas they swam mostly in straight lines in the large arena (Fig. 1F, Fig. S1D). Fish also showed straight swim patterns in a different large arena with a boundary wall (Fig. S1E). These observations indicate that the size of the behavioral arena significantly impacts the swim patterns of larval zebrafish.

### Large environment expands repertoires of swim patterns with less confinement artifact

**Fig. 2:**
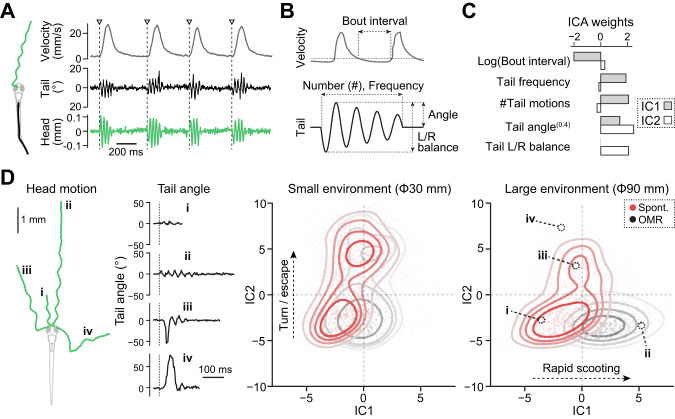
Large environment expands behavioral repertoires with less confinement artifacts. **A** *S*wim velocities (gray), tail motions (black) and head centroid motions (green) during multiple swim episodes. Triangles represent the onset of individual swim episodes. **B** Swim parameters and tail motion parameters for independent component analyses (ICA). **C** ICA weights for component #1 (gray) and component #2 (white). We used tail angle as a power of 0.4 because it best correlated with the swim distance (Fig. S2D). Swim parameters and tail motion parameters were Z-scored individually before performing ICA. **D** Scatter density plot of various swim patterns in the ICA space reveals enriched repertoires of swimming in a large environment. *Left*, head centroid trajectories and tail motions of four representative swim patterns. *Right*, scatter density plots of swim patterns in the small and large environment during spontaneous exploration (red) and optomotor response (black) in the ICA space. The same number (1200) of randomly selected swim events from *N* = 22 and *N* = 20 fish for the small and large dishes, respectively, were plotted for each condition. The loci of four representative swim patterns on the left are marked in black circles. The larger environment (90 mm) facilitated rapid long scooting (IC1) during optomotor response and fewer turnings/escapes (IC2) during both spontaneous exploration and optomotor response.

We quantified tail motions using a deep neural network (EfficientNet-B6 [44]) implemented in DeepLabCut software [45] (Fig. 2A). We trained the network over 550 images (Fig. S2A) manually annotated for ten body parts, including the eyes, nostril, body trunk, and six points along the tail. We fitted quadratic curves to the identified points along the tail to quantify tail motions (Fig. S2B). Lateral movements of the head centroids are always synchronized with the tail movements (Fig. S2C) and were used as a reference for extracting tail motion parameters (Fig. 2B). We validated the accuracy of quantified tail movements by examining how well tail parameters can predict the swim distance for each swim event. Our prediction model based on extracted tail parameters yielded a Pearson correlation coefficient of 0.89 ± 0.036 across 20 fish, indicating a highly accurate extraction of tail motion parameters (Fig. S2D).

We next examined how arena sizes affect latent behavioral states by using independent component analysis (ICA) of five separate parameters of swimming (Fig. 2C): frequencies of tail motions, angles of tail motions, the number of tail motions, the balance of tail motions between left and right sides, and intervals between swim episodes. Such dimensionality reduction analysis based on multiple parameters yields more robust estimates of latent behavioral states [46]. We applied this analysis to swim episodes in the central part of small and large arenas (Fig. 1, see “Methods”) and identified the first two independent components (IC1, IC2) in an unbiased manner (Fig. 2C). These components allowed us to map various types of swim patterns, including short scooting (i), rapid long scooting (ii), routine turns (iii), and C-turns (iv), into different loci on a low-dimensional space (Fig. 2D). IC1 separated short scooting during spontaneous exploration and long rapid scooting during optomotor response. IC2 separated scooting and turning/escape behaviors.

This mapping method showed a clear separation of swim patterns during optomotor response from those during spontaneous exploration in the large arena (Fig. 2D). Such separation was obscure in the small arena. Long rapid scooting (IC1) was dominant during optomotor response in the large arena, whereas turning and escape behaviors (IC2) were more dominant in the small arena (Fig. 2D, Fig. S2E). At the individual parameter level, the large arena allowed higher tail frequency and more tail motions per bout during visual stimulus motion and longer bout intervals during spontaneous exploration (Fig. S2F), consistent with the above observation of swim trajectories (Fig. 1). These body kinematics analyses demonstrate that a large arena, which is more than 20 times the body length of larval zebrafish, is essential for evaluating the full extent of swimming repertoires while minimizing confinement-induced turning/escape behaviors.

### Psilocybin has stimulatory effects on spontaneous exploration

**Fig. 3:**
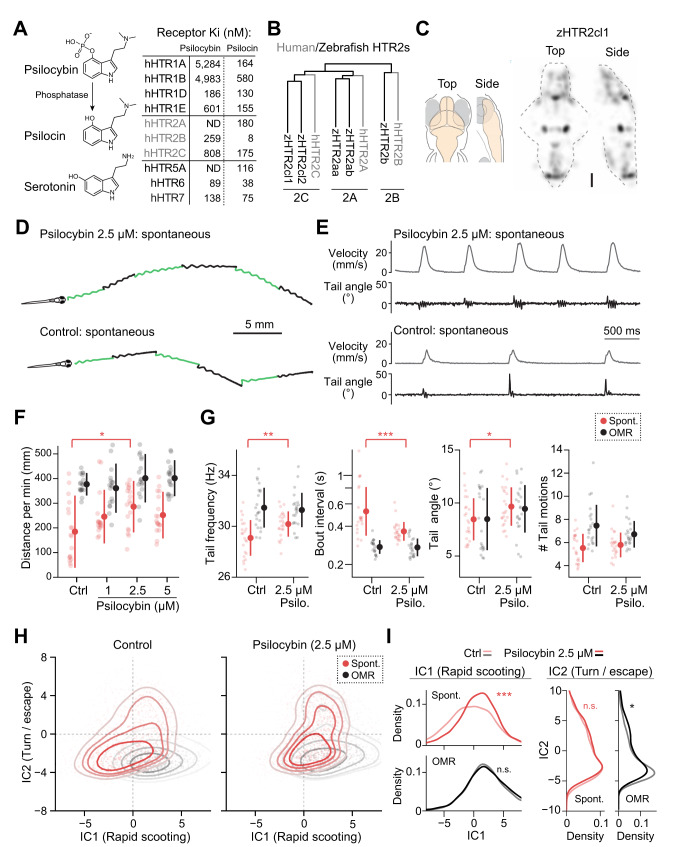
Psilocybin has stimulatory effects on spontaneous exploration. **A** Affinities of psilocybin and its metabolite psilocin to human serotonin receptors. Upon ingestion, psilocybin is metabolized by endogenous phosphatases into psilocin, which is structurally similar to serotonin. Psilocin has nanomolar affinities to a wide range of serotonin receptors. Affinities values are taken from a reference [48]. **B** Unbiased homology analysis of protein sequences revealed conserved subclasses of type 2 serotonin receptors between zebrafish and humans. **C** Average expression map of HTR2cl1 gene across 5 zebrafish brains obtained by using RNA fluorescence in situ hybridization. Scale bar, 100 μm. See “Methods” for details. **D** Head trajectories of psilocybin-treated and control fish during spontaneous exploration. **E** Psilocybin evokes rapid scooting behaviors during spontaneous exploration. **F** At a concentration of 2.5 μM, psilocybin significantly enhances swimming distances during spontaneous exploration but not during optomotor response. *N* = 18 fish for each condition. *p = 0.011 from Tukey’s post-hoc test after one-way ANOVA detected a significant difference (*F* = 3.6) among groups for spontaneous swimming distance. **G** Psilocybin significantly enhanced tail frequency, shortened bout intervals, and slightly enhanced tail angles during spontaneous swimming. *N* = 22 and 23 fish for control and 2.5 μM conditions, respectively. *P* values are from a 2-sample *t*-test between groups. **p = 0.0051 (frequency); ***p = 8.6 × 10^-4^ (interval); *p = 0.044 (angle). **H** Independent component analysis (ICA) reveals the shift of spontaneous swim patterns toward the distribution of optomotor response along the IC1 axis. **I** Psilocybin significantly enhances rapid scooting (IC1) during spontaneous exploration, while it does not cause a significant increase in turning/escape behaviors (IC2). Statistical analyses of IC1 components were performed by using kernel density 2-sample test (see “Methods”). We included 6128 (control) and 9395 (2.5 μM) swim episodes for the statistics of spontaneous exploration and 6413 (control) and 6427 (2.5 μM) swim episodes for the statistics of optomotor response. ***p = 3.3 × 10^−^^65^ and *p = 0.013 between the control group and those after exposure to 2.5 μM psilocybin. n.s., not significant (*p* > 0.05). Error bars represent standard deviations across tested fish.

We tested the effects of psilocybin treatment on spontaneous exploration and optomotor response by using the above machine learning methods. Psilocybin and its metabolite psilocin act as agonists for serotonin receptors. Upon ingestion, psilocybin converts to psilocin by the action of endogenous phosphatases. Psilocin crosses the blood-brain barrier [47] and has stronger affinities to serotonin receptors (Fig. 3A). Psilocin has affinities to multiple types of serotonin receptors, including inhibitory HTR1 receptors and excitatory HTR2 receptors [48–50]. Serotonin receptors in zebrafish are highly similar to those in humans and include major types from HTR1 to HTR7 and their subtypes. Unbiased homology analysis of protein sequences showed robust co-clustering of human and zebrafish serotonin receptors down to subtypes such as HTR2A, 2B, and 2C (Fig. 3B, Fig. S3A). We also confirmed the high expression of zebrafish HTR2 receptors in the brain (Fig. 3C). Therefore, it is reasonable to hypothesize that psilocybin and its metabolite psilocin have behavioral effects on larval zebrafish.

We found that acute, short bath pretreatment with psilocybin (2.5 μM, 4 h) in larval zebrafish had stimulatory effects on spontaneous exploration. We determined this pretreatment protocol after testing dosages between 1 μM and 50 μM and durations between 30 min to 24 h (Fig. S3B). The concentration of 2.5 μM amounts to a slightly higher dosage (0.71 mg/kg) compared to the clinical dosage in humans (0.6 mg/kg) [51]. This optimal duration of 4 h is consistent with the time course of passive diffusion of a drug with similar molecular weight into the brain of larval zebrafish [52]. We observed the reduction of such effects at higher concentrations and longer durations, indicating that the action of psilocybin saturates at this relatively low concentration compared to serotonin-selective reuptake inhibitors (see below). This saturation effect is consistent with clinical observations in human subjects [53].

After pretreatment with psilocybin, fish swam at shorter intervals with faster velocities (Fig. 3D, E), resulting in enhanced swim distance during spontaneous exploration (Fig. 3F). They showed significantly enhanced tail beat frequencies and shorter intervals between swim bouts similar to those observed during optomotor response (Fig. 3G). We did not see noticeable changes in these parameters during optomotor response, indicating that psilocybin’s effect is limited to spontaneous exploration in our paradigm. ICA analysis of swim parameters also confirmed the above observation (Fig. 3H). We observed a significant shift in spontaneous swim patterns toward the rightward direction of rapid scooting during optomotor response along the IC1 axis (Fig. 3I). These results indicate that psilocybin stimulates swim patterns in a partially similar manner to visual stimuli.

The effect of acute exposure to psilocybin was different from those of serotonin-selective reuptake inhibitors (SSRIs) that block serotonin reuptake and increase serotonin concentration at synapses. Full therapeutic effects of SSRIs do not occur during acute dosage in humans [5], and some studies showed elevated anxiety levels during the first few weeks of SSRI treatment [54]. Consistently with previous reports in zebrafish [55–57], we observed that pretreatments with fluoxetine and fluvoxamine have suppressive effects on swim patterns (Fig. S3). While fluoxetine caused noticeable distortions in the swim patterns and low-frequency tail motions, fluvoxamine caused decreases in the amplitudes of tail motions (Fig. S3C). Swimming distances decreased linearly with the dosage for both spontaneous exploration and optomotor response, indicating that SSRIs suppress motor circuits in the brain regardless of external stimuli (Fig. S3D). ICA analysis of swim parameters also showed the suppressive effect of fluoxetine and fluvoxamine as a leftward shift of behavioral states along the IC1 axis (Fig. S3E). Fluoxetine-induced distortion of swim patterns also appeared in the upper-left quadrant in the ICA space where normal swim patterns do not occupy (Fig. S3E). These differences in behavioral effects between psilocybin and SSRIs suggest that psilocybin’s stimulatory effects may occur from its selective affinities to a subset of serotonin receptors (Fig. 3A).

### Psilocybin prevents stress-induced changes in swim patterns

**Fig. 4:**
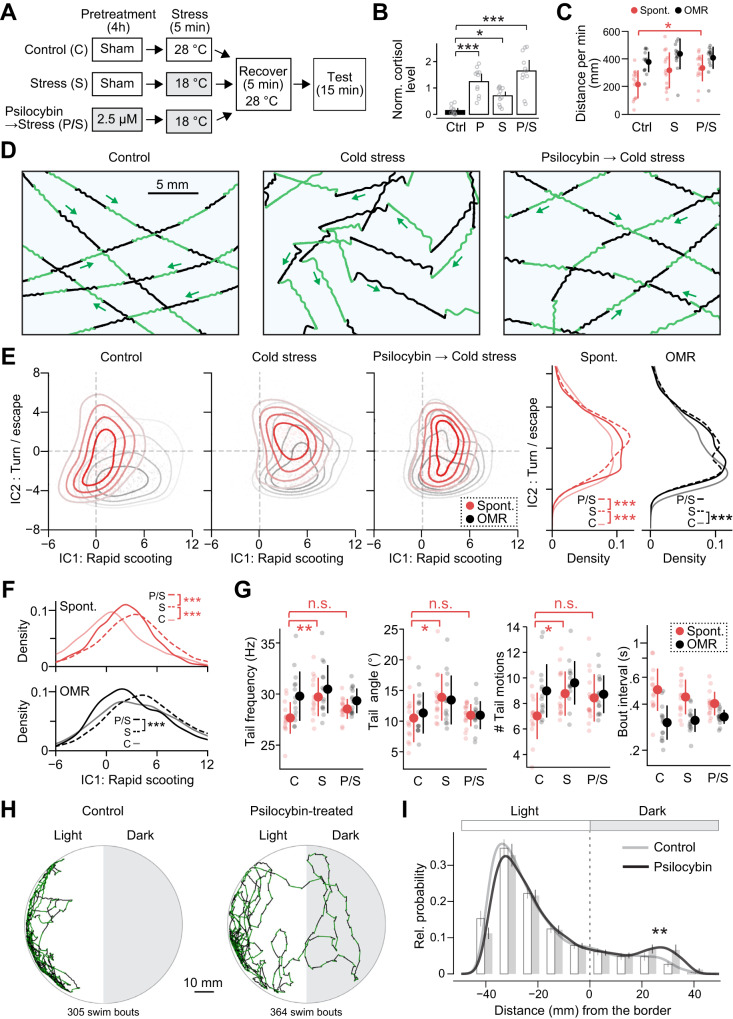
Psilocybin prevents stress-induced behavioral changes. **A** Behavioral paradigms for psilocybin treatment and acute cold shock. Shaded boxes indicate changed temperature or drug conditions. **B** Cortisol induction by stress exposure and psilocybin treatments. *N* = 11 samples for each fish. ***p = 9.6 × 10^−^^6^ and 2.1 × 10^−^^8^ for psilocybin and psilocybin/stress, respectively. *p = 0.026 for stress. We used Dunnett’s multiple comparison test against the control. **C** Swimming distances during spontaneous exploration (red) and optomotor response (black). *N* = 14 (C), 14 (S), and 15 (P/S) fish. *p = 0.021 from Tukey’s post-hoc test between the control and P/S conditions after one-way ANOVA detected significant differences among groups for spontaneous swimming distance. **D** Acute cold shock induced zig-zag swim patterns during optomotor response, and psilocybin prevented such stress-induced behavioral changes. **E** *Left*, independent component analysis (ICA) revealed the shift of swim patterns toward turn/escape behavior (IC2) after cold shock. Pretreatment with psilocybin prevented such a shift. *Right*, statistical analyses of the occurrences of turning/escape behaviors along the IC2 axis. We used kernel density 2-sample test (see “Methods”). We included 4216 (C), 3833 (S) and 4872 (P/S) swim episodes for the statistics of spontaneous exploration, and 3312 (C), 2713 (S) and 2706 (P/S) swim episodes for the statistics of optomotor response. ***p = 1.8 × 10^−^^31^ (C vs. S) and 3.0 × 10^−^^8^ (S vs. P/S) during spontaneous exploration. ***p = 1.0 × 10^−^^7^ (C vs. S) during optomotor response. **F** Statistical analyses of the stimulatory effect along the IC1 axis. ***p = below 1.0 × 10^−^^200^ (C vs. S) and 1.7 × 10^−^^35^ (S vs. P/S) during spontaneous exploration. ***p = 4.8 × 10^−^^5^ (S vs. P/S) during optomotor response. **G** Analyses of individual swim parameters. Acute cold shock significantly increased tail frequency, tail angle and the number of tail motions, while such an effect was not observed in fish pretreated with psilocybin. Statistical tests used Tukey’s post-hoc test after one-way ANOVA among different conditions during spontaneous explorations: **p = 0.0035 (tail frequency); *p = 0.032 (tail angle); *p = 0.046 (tail motions). Error bars represent standard deviations across tested fish. **H, I** Psilocybin ameliorates dark-avoidance behavior in larval zebrafish. *H*, representative swimming trajectories of the control and psilocybin-treated fish during a 5-min spontaneous exploration in an environment with light and dark areas. The numbers of plotted swim events are shown at the bottom of the panel. *I*, spatial distribution of swim events relative to the border between the light and dark areas in control (gray) and psilocybin-treated (black) fish. We analyzed 23,103 and 27,303 swim events from 30 control and 30 psilocybin-treated fish, respectively. Density plots, as well as binned histograms (9-mm intervals) from individual fish, were plotted. Error bars represent the standard error of the mean (s.e.m.) across the tested fish. **p = 0.0082 from kernel density 2-sample test between the control and psilocybin-treated fish.

Acute administration of psilocybin has anxiolytic effects in humans [58, 59]. We thus tested whether acute stress exposure changes fish’s swim patterns in our setup and whether psilocybin can prevent such stress-induced behavioral changes. Various environmental stressors have been tested in larval zebrafish that trigger cortisol increase, including hypertonic water [35,60–66], acids [60], mechanical disturbance [35, 64, 66, 67], social isolation [66], and heating [66, 68] / cooling [66] shock. Here we used a cold shock paradigm that rapidly lowers the temperature by 10 degrees (28 to 18**°**C, Fig. 4A) as it is least likely to cause lasting changes in tissue integrities, protein folding, and ionic balance in the body [66]. We also tested the effect of hypertonic stress for comparison (Fig. S4). We pre-treated fish with psilocybin with the most effective concentration for enhancing spontaneous exploration (2.5 μM, Fig. 3), exposed them to stressors for 5 min, recovered them at a normal temperature, and tested their spontaneous exploration and optomotor response (Fig. 4A). We quantified cortisol levels induced by the cold shock paradigm using enzyme-linked immunosorbent assay (Fig. 4B, Fig. S3F). Cold shock paradigm significantly increased cortisol in larval zebrafish, and, interestingly, psilocybin pretreatment per se also induced comparable amounts of cortisol. Psilocybin pretreatment and the cold shock paradigm had additive effects on cortisol induction. This observation is consistent with the induction of cortisol in humans [59] and corticosterone in rodents [69] after acute administration of psilocybin. The swimming distance also increased after exposure to both cold and hypertonic stressors (Fig. 4C and Fig. S4A), which is consistent with previous zebrafish studies [60, 65] and demonstrates the robustness of our stress protocol.

We found that acute stress exposure caused “zig-zag” swimming patterns compared to the control (Fig. 4D). During optomotor response, the control fish show straight trajectories in our large arena (Fig. 1F). Such patterns changed into zig-zag patterns, as each bout started from a sharp turning of the head and large tail undulation to one side (pattern [iii] in Fig. 2D) instead of smooth scooting (patterns [i] or [ii] in Fig. 2D). This type of change was also observed after exposure to hypertonic stress (Fig. S4B), suggesting that the emergence of zig-zag swim patterns, as compared to normal smooth straight patterns, can be a robust indicator of stress-induced behavioral changes in larval zebrafish.

Importantly, pre-treatment with psilocybin prevented stress-induced changes in swim patterns. Psilocybin-pretreated fish exhibited straight swim patterns even after the stress exposure (Fig. 4D). This prevention of stress-induced behavioral changes was also evident in the ICA analysis along the axis of IC2, which represents occurrences of escape/turning behavior (Fig. 4E). Acute cold shock significantly elevated distributions along the IC2 axis (Fig. 4E). The pretreatment with psilocybin significantly diminished the occurrence of turning/escape behavior after cold shock (Fig. 4E). Notably, psilocybin pretreatment only partially reversed the stress-induced shift along the IC1 axis, which represents occurrences of rapid scooting behavior (Fig. 4F). This remaining shift along the IC1 axis is consistent with the stimulatory effect of psilocybin pretreatment per se (Fig. 3G). The preventative effect of psilocybin was also evident in individual tail kinematics, such as frequencies and angles of tail motions (Fig. 4G). These results suggest that psilocybin prevents the stress-induced occurrence of escape/turn behavior while still exerting its stimulatory effect.

The preventative effects of psilocybin were not observed for behavioral changes induced by hypertonic stress (Fig. S4C, D). Previous studies showed that cold shock and hyperosmotic stress trigger different types of cardiac responses in zebrafish [70] and that acute hyperosmotic stress may leave lasting damage to the tissue integrities [71]. Therefore, it is possible that hyperosmotic stress incurred persistent damages in zebrafish larvae that cannot be reversed pharmacologically. Nonetheless, this observation demonstrates that the reversal of stress-induced behavioral shifts along the IC2 axis does not simply occur from the induction of straight swim patterns at the motor circuit level.

We also examined whether psilocybin mitigates the innate anxiety response of larval zebrafish by measuring the dark avoidance behavior (Fig. 4H). We found that psilocybin-treated fish explore the darker side significantly more often than the control fish (Fig. 4I). This result indicates that psilocybin can ameliorate both innate and externally induced anxiety responses.

We further examined the involvement of HTR2 receptors in stress-induced behavioral changes by using ketanserin, an HTR2 receptor antagonist [72], which also inhibits monoamine transporters [73] and histamine receptors [74]. Consistent with studies that showed its anxiogenic effects in zebrafish [75, 76], we found that bath application of ketanserin significantly shifted behavioral states along the IC2 axis in a similar manner to cold shock and that psilocybin prevented such changes (Fig. S4E, F). These results suggest the crucial role of HTR2 receptor pathways in behavioral changes following stress exposure and demonstrate that the observed stimulatory and anxiolytic effects of psilocybin likely occur from the modulation of such endogenous serotonergic pathways in the zebrafish brain.

### Comparison with ketamine and SSRI

**Fig. 5:**
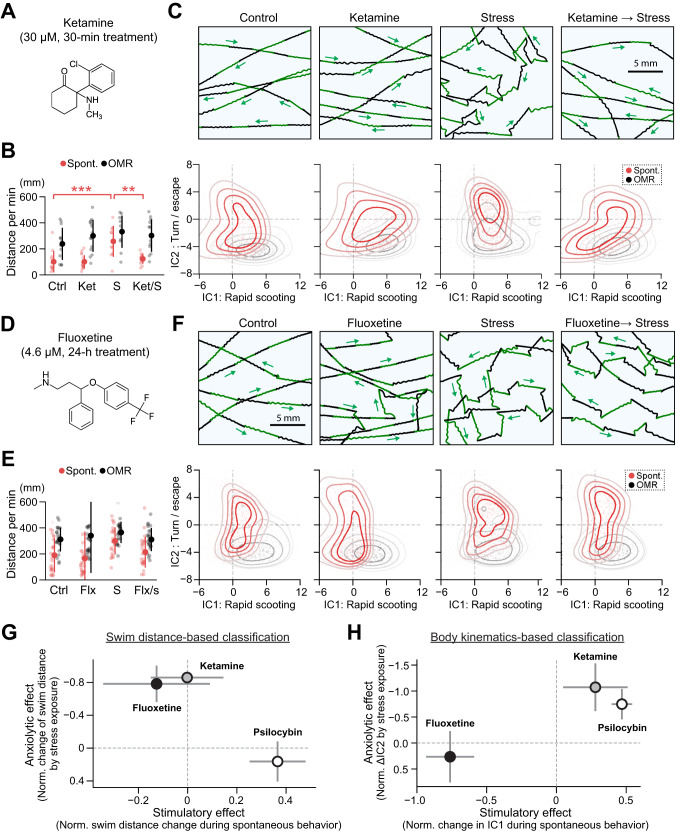
Comparison with fast-acting and slow-acting antidepressants. **A** Chemical structure and dosage of ketamine (Ket). **B** Swimming distances during spontaneous exploration (red) and optomotor response (black). *N* = 12 (C), 14 (Ket), 10 (S) and 12 (Ket/S) fish. ***p = 1.4 × 10^−^^4^ (C vs. S) and **p = 1.7 × 10^−^^3^ (S vs. Ket/S) from Tukey’s post-hoc test after one-way ANOVA for spontaneous swimming distance. **C** *Top*, acute cold shock induced zig-zag swim patterns during optomotor response, and pre-exposure to ketamine prevented such stress-induced behavioral changes. *Bottom*, independent component analysis (ICA) revealed the shift of swim patterns toward turn/escape behavior (IC2) after cold shock, which was prevented by ketamine. See Fig. S5A for statistical analyses. **D** Chemical structure and dosage of fluoxetine (Flx). **E** Swimming distances during spontaneous exploration (red) and optomotor response (black). *N* = 24 (C), 24 (Flx), 25 (S) and 24 (Flx/S) fish. **F** *Top*, acute cold shock induced zig-zag swim patterns during optomotor response, and pre-exposure to fluoxetine did not prevent such stress-induced behavioral changes. *Bottom*, independent component analysis (ICA) revealed the shift of swim patterns toward turn/escape behavior (IC2) after cold shock. Pretreatment with fluoxetine did not prevent such a shift. See Supplementary Fig. S5B for statistical analyses. **G** Summary of the behavioral effects of tested antidepressants estimated from changes in spontaneous swimming distances. Stimulatory effects (horizontal axis) were estimated from the change of swimming distance after drug administrations compared to the average of control fish. Anxiolytic effects (vertical axis) were estimated from how much the drug prevented the stress-induced change of spontaneous swimming distance compared to untreated conditions. Error bars represent the standard error of the mean (s.e.m.) across drug-treated fish. See “Methods” for details. **H** Summary plot of the behavioral effect of tested antidepressants estimated from changes in independent components (IC1, IC2) of body kinematics. Stimulatory effects (horizontal axis) were estimated from the changes along the IC1 axis during spontaneous swimming after drug treatment compared to the average of control fish. Anxiolytic effects (vertical axis) were estimated from how much the drug prevented the stress-induced changes along the IC2 axis compared to untreated conditions during spontaneous swimming. See “Methods” for details.

We next investigated the behavioral effects of other antidepressants and compared them with those of psilocybin. We first tested the effect of ketamine, a fast-acting antidepressant that can also suppress stress-induced behavioral changes in zebrafish [77, 78]. We used a sub-anesthetic concentration (30 µM) and performed bath application for 30 min before the cold shock (Fig. 5A, Fig. S5A). Unlike psilocybin, ketamine did not increase spontaneous swimming distance and reversed the stress-induced increase in swimming distance (Fig. 5B). However, ketamine prevented the emergence of zig-zag swim patterns after cold shock and recovered straight swim patterns (Fig. 5C). In the ICA analysis, ketamine significantly reversed the stress-induced shift along the IC2 axis (Fig. S5A). These behavioral effects of ketamine demonstrate that our behavioral analysis based on ICA of body kinematics generalizes to the effect of other anxiolytic drugs and that psilocybin has a similar acute anxiolytic effect as ketamine.

We also tested the effect of fluoxetine in the cold shock paradigm. We used a concentration of 4.6 µM from previous zebrafish studies [55, 57], and examined the effect of bath application for 24 h before the cold shock (Fig. 5D, Fig. S5B). Similar to ketamine, fluoxetine did not increase spontaneous swimming distance and reversed the stress-induced increase in swimming distance (Fig. 5E). However, fluoxetine had mixed effects on body kinematics after stress exposure. It partially reversed and partially exacerbated shifts of behavioral states along the IC2 axis (Fig. 5F). These mixed effects may be due to the induction of distorted swim patterns by fluoxetine that we observed (Fig. 5F, Fig. S3C) but also may reflect its lack of acute anxiolytic effects in humans [5].

We classified the effects of psilocybin, ketamine and fluoxetine based on two behavioral measures: changes in swimming distances (Fig. 5G) and the ICA analysis of body kinematics (Fig. 5H). In the first type of classification (Fig. 5G), we classified the action of tested drugs based solely on swimming distances. Stimulatory/suppressive effects were defined as changes in spontaneous swimming distance in unstressed fish by the application of the drugs compared to the control condition. Anxiolytic effects were calculated based on how much the drugs could revert the increase of swimming distance induced by stress exposure. In the second type of classification (Fig. 5H), we used two dimensions identified by the ICA analysis of body kinematics. Stimulatory/suppressive effects were defined as shifts along the IC1 axis in unstressed fish by the application of the drugs compared to the control condition. Anxiolytic effects were calculated based on how much the drugs could revert the changes along the IC2 axis induced by stress exposure (see “Methods” for details).

We compared the results of these two types of classifications. Their stimulatory/suppressive effects on basal behavior are consistent between these two measures, where fluoxetine is suppressive and psilocybin is stimulatory. However, their effects on stress-induced behavioral changes were different. The reversal of the stress-induced increases in swim distances (Fig. 5G) only occurred with ketamine and fluoxetine. In contrast, the reversal of the stress-induced changes in body kinematics (Fig. 5H) only occurred with ketamine and psilocybin. These results demonstrate that the use of a single behavioral indicator such as swimming distance may confound stimulatory/suppressive effects and anxiolytic effects of antidepressants and that dimensionality reduction analysis based on multiple body kinematic parameters provides a more accurate measure of their anxiolytic effects.

### Psilocybin modulates the activity of the serotonergic system

**Fig. 6:**
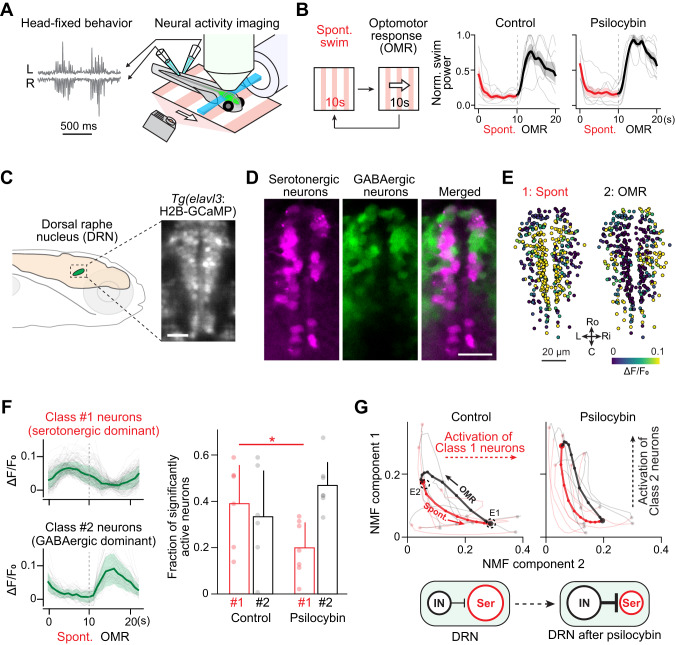
Psilocybin modulates the activity of the serotonergic system. **A** Schematics of neural activity imaging experiments. Fish is immobilized in an imaging chamber and motor signals from the tail were recorded using a pair of electrodes. A light-sheet microscope scans the brain of transgenic zebrafish that expresses genetically encoded calcium indicators pan-neuronally. A visual stimulus (red) is projected beneath the fish. **B** *Left*, behavioral paradigm. Visual gratings stop for 10 s during the spontaneous period (Spont.) and move forward for 10 s to induce optomotor response (OMR) during the OMR period. *Right*, trial-averaged swim patterns of control (*N* = 6) and psilocybin-treated fish (*N* = 7). **C** The location of the dorsal raphe nucleus (DRN) in the zebrafish brain and its image of expressing nuclear-localized calcium indicator. Scale bar, 20 µm. **D** Spatial distributions of serotonergic neurons (magenta) and GABAergic neurons (green) in the DRN. Serotonergic neurons occupy the midline area of the DRN, while GABAergic neurons exist in a more lateral area. See a referenced paper [26] for details. Scale bar, 20 µm. **E** Spatial distribution of neurons that show higher activity during the spontaneous period (left) and the OMR period (right) in a representative fish. Differences in ΔF/F between these two task periods are color-coded for each neuron. **F** *Left*, trial-averaged activity patterns of neurons activated during the spontaneous period (Class 1 neurons, red) and those activated during the OMR period (Class 2 neurons, black) from a representative fish. *Right*, fractions of significantly active neurons during the spontaneous period (red) and the OMR period (black) in control and psilocybin-treated fish. *N* = 6 and 7 for control and psilocybin-treated fish, respectively. *p = 0.046 for neurons activated during the spontaneous period by Wilcoxon’s rank-sum test. **G** *Top*, low dimensional representation of neural state dynamics in control fish (left) and psilocybin-treated fish (right). We applied non-negative matrix factorization (NMF) to the trial-averaged neural activity of all recorded neurons from all tested fish to calculate NMF components. Averaged trajectories of control and psilocybin-treated fish are plotted with thick lines, and trajectories of individual fish are plotted with thin, transparent lines. Trajectories during the spontaneous period (see B) were plotted in red, and those during the OMR period were plotted in black. Dots represent time points of recording at 1 Hz. *Bottom*, our hypothesis of psilocybin-induced changes in neural dynamics in the DRN. Psilocybin elevates the excitability of GABAergic neurons, which in turn suppresses the activity of serotonergic neurons.

Lastly, we investigated whether psilocybin affects neural dynamics in the brain of larval zebrafish by examining the neural population dynamics in the dorsal raphe nucleus (DRN). Acute administration of lysergic acid diethylamide (LSD, HTR2 agonist) and psilocin both induced rapid suppression of serotonergic neurons in the DRN in mammals [18, 19]. We investigated whether psilocybin induces similar changes in zebrafish by using a head-fixed virtual reality setup and calcium imaging of neural activity (Fig. 6A). In this setup, an immobilized zebrafish larva is placed in an imaging chamber. We record swim signals from spinal motoneurons by using a pair of electrodes attached to the tail while we project moving visual stimuli beneath the fish. Both control and psilocybin-treated fish stopped swimming or showed only occasional spontaneous swimming when the visual stimuli stopped (“Spontaneous” period), and both showed vigorous swimming when the visual stimuli moved forward (“OMR” period) (Fig. 6B). We recorded neural activity in the DRN by using our bespoke light-sheet microscope [79] during these two task periods using transgenic zebrafish that express nuclear-localized calcium indicators pan-neuronally (Fig. 6C).

The dorsal raphe nucleus in larval zebrafish mainly consists of two neural populations: serotonergic neurons that mainly reside along the midline and GABAergic neurons that mainly reside in the lateral part (Fig. 6D). Our previous study demonstrated that serotonergic and GABAergic neurons in the DRN have complementary activity patterns, where the former population respond to pause of swimming or backward optic flow during swimming, and the latter population encodes the strength of swimming [26]. Consistently, we found that neurons along the midline (predominantly serotonergic neurons) showed higher activity during the spontaneous period when the fish mostly stopped swimming. Neurons in the lateral part of the DRN (predominantly GABAergic neurons) showed higher activity during the OMR period when the fish showed vigorous swimming (Fig. 6E).

We then investigated how psilocybin treatment changes such neural population dynamics in the DRN. The fraction of neurons that showed higher activity during the spontaneous period (Class 1 neurons), which are predominantly serotonergic, became significantly lower after psilocybin treatments in the DRN (Fig. 6F). On the contrary, the fraction of neurons that showed higher activity during the OMR period (Class 2 neurons), which are predominantly GABAergic, became slightly higher. These results suggest that acute psilocybin exposure inhibits serotonergic neurons in the zebrafish DRN, potentially by activating nearby GABAergic neurons.

To visualize the shifts in such complementary dynamics between different neural populations, we applied non-negative matrix factorization (NMF) to the trial-averaged activity of all neurons in the DRN and plotted the transition of neural states based on the first two identified components (Fig. 6G). The non-negative constraint of this dimensionality reduction method [80] is suited to separate complementary activity patterns of two neural populations. We pooled trial-averaged activity patterns of all neurons in the dorsal part of the DRN from all tested fish and fit NMF (components = 2) to obtain two weights of each neuron for two non-negative population vectors. We then calculated the vectorial inner products of neuronal weights and their mean ΔF/F values for each time point in the task within each fish (thin lines) or from each group of fish (thick lines) to show neural state transitions between two components. In the control fish, neural states in the DRN shift rightward along the horizontal axis (NMF #2) during the spontaneous period by the activation of Class 1 neurons. Fish’s vigorous swimming during the OMR period shifts up the neural state along the vertical axis (NMF #1). Psilocybin treatment diminished the shift of the neural state along the horizontal axis and instead enhanced its shift along the vertical axis. These results show that psilocybin suppresses neural activations in the DRN during the pause of swimming, which indicates the suppression of serotonergic neurons (Fig. 6G).

The resemblance of this phenomenon with mammalian observations [18, 19] suggests that psilocybin triggers similar changes in neural dynamics in brain structures evolutionarily conserved between teleosts and mammals.

## Discussion

In this study, we developed a high-resolution tracking system and a machine-learning framework for evaluating how psilocybin changes the latent behavioral states of larval zebrafish. Psilocybin has stimulatory effects on spontaneous exploration and preventative effects for stress-induced behavioral changes, indicating that psilocybin induces unique neural dynamics that are both stimulatory and anxiolytic. Neural activity imaging in the dorsal raphe nucleus suggested that psilocybin suppresses the activity of serotonergic neurons. These observations have similarities with those in past mammalian studies, indicating the presence of common neural mechanisms in the evolutionarily conserved brain areas through which psilocybin exerts its behavioral effects.

Tracking precise body kinematics in a larger environment was critical for our findings in this study. Our 90-mm arena facilitated straight swim patterns and suppressed turning/escape behaviors compared to a small arena, and it allowed us to identify distinct behavioral states that affect spontaneous exploration, visually-driven rapid scooting and irregular swim patterns after stress exposure. These results indicate that high-throughput assays in small arenas such as multiwell plates may impair the full spectrum of the fish’s behavioral repertoires. Moreover, we demonstrated that environmental stimuli that evoke different types of body kinematics, such as moving visual stimuli and acute stress exposure, could yield similar changes in macroscopic locomotion measures such as swim distance per minute (Fig. 1, Fig. 4, Fig. 5 and Fig. S4). Precise tracking of multiple body kinematic parameters and machine learning inference of latent behavioral states enabled separate evaluation of stimulatory/suppressive effects and anxiety effects, and such separation was critical for comparing anxiolytic effects of multiple antidepressants drugs (Fig. 5)

Our observations open up new opportunities for further investigations into neural mechanisms by which psilocybin affects behaviors. While psilocybin is known primarily as an agonist for type 2 serotonin receptors, it may also activate HTR1 receptors to exert its behavioral effects [49, 50]. Type 1 receptors are densely expressed in the brainstem areas of mammals [81, 82] and zebrafish [83], whereas type 2 receptors are densely expressed in neocortical, cerebellar and pontine areas of mammals [17, 84, 85] and zebrafish [86]. Therefore, psilocybin likely acts on these receptors to alter neural dynamics in the brain and behavior of zebrafish.

How does psilocybin stimulate swimming in a partially similar manner to visual stimuli? Neural mechanisms that trigger spontaneous swimming remain mostly elusive in zebrafish. Recent studies found that spontaneous activation of a sensory neural ensemble in the optic tectum triggers spontaneous swimming [87]. Therefore, it is possible that psilocybin stimulates a part of the sensorimotor reflex circuit to induce swim patterns that are partially similar to those during optomotor response (Fig. 3). It is also possible that persistent activation/suppression of motor circuits underlies such behavioral changes. Serotonergic neurons in the dorsal raphe nucleus have suppressive effects on zebrafish behavior [26, 28], and the suppression of their activity by psilocybin (Fig. 6) may stimulate behavior. Further investigation into brain-wide neural dynamics based on large-scale neural activity imaging methods [20, 79] and histological neural activity mapping methods [32] will be necessary to disambiguate these potential mechanisms.

Psilocybin’s preventative effects for stress-induced behavioral changes may occur from the same neural mechanisms responsible for its stimulatory effect on spontaneous explorations, as we found that both seem to bring the fish’s swim patterns toward an intermediate state between spontaneous exploration and visually-driven optomotor response. Such common mechanisms can occur at neural circuit levels and molecular levels [12, 88]. Acute administration of psilocybin increased cortisol in larval zebrafish (Fig. 4B), consistent with observations in mammals [59, 69]. Such a paradoxical effect on cortisol indicates that the increase in cortisol levels is not directly causal to the observed changes in swim patterns after stress exposure. It will be necessary to determine whether psilocybin prevents stress-induced behavioral changes by inducing cortisol or other neural mechanisms irrelevant to the action of the hypothalamic-pituitary-adrenal axis.

This study only focused on the acute effect of psilocybin in larval zebrafish and did not address other clinically relevant actions of psilocybin. For example, psilocybin and other HTR2 agonists are effective in reversing depression-like behaviors after chronic stress exposure in rodents [15–17] and humans [9, 10], and a single dose has lasting effects [9]. The latter persistent effect is unique to psilocybin compared to other antidepressants such as ketamine and SSRIs, but its underlying mechanisms are largely unknown. Our findings pave the way to examine serotonergic psychedelics’ unique pharmacological actions in the brains of larval zebrafish, which allow for live tracking of neural activity [20, 26, 28], neurotransmitters [89, 90], structural plasticity [91], and molecular dynamics across the brain [92].

## Methods

### Animal experiments

All of the experiments in this study that use zebrafish larvae were performed under the approval of the Institutional Animal Care and Use Committee (IACUC) and the Institutional Biosafety Committee (IBC) of the Weizmann Institute of Science and by the Israeli National Law for the Protection of Animals - Experiments with Animals (1994). The use of psilocybin in our research was conducted under the supervision of the Israel Ministry of Health (number # 984.02.2022) and the IACUC (number # 07550922-2) of the Weizmann Institute of Science.

### The hardware of the zebrafish tracking system

We used a custom-built zebrafish tracking system consisting of a high-speed camera (FLIR, ORX-10G-51S5M-C monochromatic), a macro lens and its locking sleeve (ZOOM 7000 & 1-11736, Navitor), an infrared filter for the lens (Hoya R-72, Edmund #54-753), 880 nm LED illumination (100 × 200 mm, BL040801-880-IC, Advanced Illumination; Edmund # 66-844), a power supply and manual intensity adjustments for the LED (Edmund # 86-887, #66-855), a 100 × 120 mm cold mirror (Edmund #64-452), and a compact projector (Optoma LV130). Structural parts that hold these devices were purchased from Thorlabs or manufactured by the Physics Core Facility of the Weizmann Institute of Science.

The acquisition PC was equipped with a 10 G Ethernet adapter (Intel X540-T2) for data transfer from the camera and an NVMe disk drive that has a 15-terabyte capacity and >3GB/s writing speed (Micron 9300). A custom-written Python application based on PyQT5 (https://pypi.org/project/PyQt5/) and pyqtgraph libraries (https://www.pyqtgraph.org/) controlled devices and acquired data from the camera. Many modules of the application were inherited from PyZebrascope software [79] we previously developed for light-sheet microscopy. Communication and data acquisition with the camera used PySpin library (https://pypi.org/project/pyspin/). Raw 8-bit data of the acquired image was saved in a single AVI file for each experiment using OpenCV-Python library (https://pypi.org/project/opencv-python/).

We tested the behavior of *AB* fish at the age of 5 days post fertilization (dpf), except for experiments with SSRIs that were tested at 6 dpf, on a chemical watch glass (125 mm, AlexRed) whose bottom surface was manually coated with a white spray. We imaged an area that spanned 90 mm (1100–1200 pixels) for each dimension with a resolution of 83 μm per pixel at 290 Hz. We recorded the behavior of each fish (one fish per experiment) for 15 min, which resulted in >250,000 frames and ~300 GB file size for each experiment. We excluded fish which showed less than 50 swim events (6.7 swims per minute) during the period of optomotor response. Behavioral data presented in this study were acquired using at least three batches (Figs. 1–5, Fig. S1D, Fig. S2D, E, F, Fig. S3D, E, Fig. S4, Fig. S5) except the dose optimization of psilocybin using at least two batches (Fig. S3B).

### Pharmacological treatments

Until 5 dpf, both control and drug-exposed embryos were reared in 90-mm Petri dishes (Miniplast, 820-090-01-017) and maintained in a light-cycled incubator at 28.0 °C. Media was changed every other day and no food was given before behavioral experiments. All behavioral experiments in this study were performed in E3 medium after washing out pretreated drugs.

#### Psilocybin administration

Psilocybin solution (Sigma, P-097) was purchased as a stock solution concentration of 1.0 mg/mL in acetonitrile:water (1:1), ampule of 1 mL (3.52 mM). Stock solutions were stored at −80 °C for long-term storage, and in-use aliquots were stored at −20 °C. Aliquots were thawed and vortexed immediately before use. Psilocybin was administered by incubating the fish in the psilocybin solution in 6-well plates (Corning, #3516). Desired concentrations of psilocybin were achieved by adding the stock solution to the E3 medium in which the fish swim. Concentrations ranging from 1 to 50 μM were tested in order to optimize the dosage. We determined that a dose of 2.5 μM for 4 h had the largest effect on spontaneous swimming distance compared to controls (Fig. 3F, Fig. S3B). Following the 4-h exposure, fish were double washed in E3 medium and remained in E3 medium until their behavior was examined. Behaviors were recorded for 15 min per fish.

#### SSRI administration

Fluoxetine hydrochloride (Sigma, F918 or Abcam, ab120077) was prepared as a 1 mg/ml (2.9 mM) stock solution in a conditioned E3 medium for zebrafish embryos. Fluvoxamine (Sigma, F2802) was prepared as a 10 mg/ml (23 mM) stock solution in a conditioned E3 medium. Desired Fluoxetine and Fluvoxamine concentrations were achieved by diluting the stock solution with conditioned E3 medium and storing the aliquots at −20 °C. At 5 dpf the fish were incubated as follows: fluoxetine and fluvoxamine were administered by incubating the fish in the solutions in 6-well plates. Desired concentrations of the two types of SSRIs were achieved by adding the stock solutions to the E3 medium in which the fish swim. Concentrations ranging from 1 to 10 μM for Fluoxetine and 2.5 to 25 μM for Fluvoxamine were tested in order to optimize the dosage. After 24 h of incubation the fish, after being triple washed, were transferred to a new Petri dish, similar to the one they dwelled in before, containing only E3 medium. Behaviors were recorded at 6 dpf for 15 min per fish.

#### Ketamine administration

Ketamine hydrochloride solution (Zoetis, Ketavet 100 mg/ml) was diluted with E3 medium to the concentration of 30 µM just before the experiments. Ketamine was administered by incubating the 5-dpf fish in the solutions in 6-well plates. After 30 min of incubation, after being triple washed, fish were transferred to a new Petri dish, similar to the one they dwelled in before, containing only E3 medium. To prevent potential effect of stress due to solution exchanges and washing, the fish were recovered for 20 min before further going through the cold shock paradigm. Behaviors were recorded at 5 dpf for 15 min per fish.

#### Stress paradigms

We utilized two established larval zebrafish stress paradigms to induce stress in the fish; hyperosmotic stress and cold temperature stress.

Zebrafish are freshwater fish, and thus, a salt-water environment induces psychological and physiological stress. In order to create an osmotic environment, NaCl was dissolved in E3 medium in concentrations of 25, 50, and 100 mM NaCl, which have previously been found to induce stress in zebrafish [35] (Fig. S4A). Fish were placed in the osmotic solution for 15 min and were then triple-washed immediately before behavioral recordings.

Just as osmotic changes to the water cause stress to the fish, changes in water temperature are also known to induce stress. Zebrafish’s optimal environment is approximately 28 °C, and so it has been shown that a short exposure of 5 min to 18 °C leads to increased cortisol levels and anxiety-like behaviors in larval zebrafish [66]. Thus, we utilized this established stress paradigm and exposed the fish to 18 °C E3 medium for 5 min. We then returned the fish to 28 °C E3 for a 5-min recovery before testing (Fig. 4A). 18 °C E3 was achieved by mixing 4 °C E3 with 28 °C E3. Over the course of the 5-min cold stimulus, the water temperature, on average, ranged from 18 to 20 °C. The recovery in 28 °C E3 was done in an incubator; thus, the temperature remained stable. Note that these procedures involve multiple occasions of transferring fish and replacing liquid around the fish, which themselves cause stress response due to mechanical disturbances. The effect of such procedural stress was present as the shifts of behavioral states along the IC2 axis in the data presented in Fig. 4 and Fig. S4 compared to other datasets.

#### Ketanserin administration

For ketanserin experiments (Fig. S4E), we performed a bath application of ketanserin (11.25 μM) for 5 h before behavioral tests. Psilocybin (2.5 μM) was added to the solution 1 h after the start of ketanserin treatment for a total duration of 4 h before behavioral tests. Ketanserin (Sigma, S006) was prepared as a 0.5 mg/mL stock solution in E3 medium. Stock solutions were stored at −80 °C for long-term storage, and in-use aliquots were stored at −20 °C. The desired concentration of ketanserin was achieved by adding the stock solution to the E3 medium in which the fish swim.

### Tracking zebrafish and its tail motion

We used custom Python scripts to extract swimming parameters and tail movements. We applied the following procedures to each data.

In the first step, we identified the pixel-level centroid position of the head of the fish for each frame and extracted square patches around the fish. To do this, we calculated the average background image based on 100 images equidistantly sampled from all time points and subtracted it from the movie. We applied a Gaussian blur filter (σ = 250 μm) to background-subtracted images and identified the darkest pixel as the centroid position of the head of the fish. We cropped the image (144 by 144 pixels, 12 × 12 mm) around the centroid, rescaled it to between 0 and 255 brightness values, and stored them in a separate AVI file. To accelerate file processing time, we used a recurrent algorithm that searched proximities of the fish positions of previous time points.

In the second step, we automatically annotated body parts from the extracted fish images by using a deep neural network (EfficientNet-B6) implemented in the DeepLabCut package [45]. We trained the network by using 550 manually annotated images (Fig. S2A). During the development process, we observed that biases in the training datasets were reflected in the automatic annotation results. Therefore we balanced the repertoires of training images so that they covered all angles of the tail symmetrically between the left and right sides. We also included images with outlier pixels, which result from a small inhomogeneity of the bottom coating of the dish. Errors in manual annotations were further screened by independent algorithms that detected significant deviations in distances between annotated body parts and manually corrected them. The Deep neural network was trained for 30,000 iterations with the “imgaug” augmentation option. The average test error was 1.47 pixels.

In the third step, we applied [i] subpixel head centroid detection and [ii] tail angle quantification for each frame. For subpixel head centroid detection (Fig. S1C), we applied a Gaussian blur filter to the fish image and applied a subpixel centroid detection algorithm implemented in Photutils package (https://photutils.readthedocs.io/) originally developed for detecting subpixel motions of cosmic stars in telescopic images. This identification of subpixel-level centroid position was critical for visualizing swim trajectories throughout this study and also for extracting tail motion parameters during swim bouts. For tail angle quantification (Fig. S2B), we fitted a quadratic function to seven annotated points along the body trunk and the tail and quantified the angle of the fit function relative to the body-nostril axis. The angle quantification was made at 1 mm from the base part of the tail. We tried several other methods for tail angle quantifications from previous studies and found that quadratic fit provides the best signal-to-noise ratio in this low-resolution imaging system.

In the last step, we identified each swim bout based on swim velocities and extracted  basic swim parameters (position, velocity, duration, moved distance, etc.) and tail kinematic parameters (frequency, number, angles of tail motions). We found that lateral motions of subpixel head centroid relative to the direction of the swimming, which synchronously precedes tail motions, provide a slightly better signal-to-noise ratio for defining individual cycles of tail motions in this low-resolution imaging system. Therefore, our algorithm used the motion of the subpixel head centroid as a reference to read out maximum tail angles for each tail motion cycle (Fig. S2C). Peaks of tail angles are detected for each tail motion cycle and further averaged across cycles to quantify average tail angles. Tail frequencies are determined based on these tail motion cycles. The left / right (L/R) balance of the tail motion is calculated by using the below equation

$$L/R\,{Balance}=\left|\frac{\varSigma ({Right}\,{tail}\,{angles})-\varSigma ({Left}\,{tail}\,{angles})}{\varSigma ({Right}\,{tail}\,{angles})+\varSigma ({Left}\,{tail}\,{angles})}\right|$$

so that the value becomes 0 if the tail motions are symmetric and 1 if there is only one tail movement to a specific side. We tested the accuracy of tail motion tracking by examining how accurately we can predict the swim distances from tail motion parameters (Fig. S2D). We created a multiplicative model that predicts the distance of swimming based on the frequency, number, and average angle of tail motions for each swim bout. We extracted forward swim events that occurred in the central part of the large dish (30 mm from the center) and caused changes in the head direction less than 30 degrees. We fit the model by using the L-BFGS-B method implemented in the minimize function of Scipy package and identified optimal values for the power factors for each tail parameter across 20 fish. The optimal power factors were roughly 0.4 for the tail angle and 1 for the frequency and the number of tail motions. We then calculated the correlation coefficient with the actual swim distance and the predicted distance from tail motions (Fig. S2D).

### Data analysis of tail motions

We used custom Python scripts to summarize the results presented in this study. Except for the analysis of swimming distances (Fig. 1D, Fig. 3F, Fig. 4C, Fig. 5B, E, G, Fig. S3B, D and Fig. S4A), we only included swim episodes that occurred in the central part of behavioral arenas (within 10 or 30 mm from the center of the small or large arena, respectively) for the analyses of swim/tail parameters throughout this study to rule out the physical effect of the wall in the small arena and shallow places in the large arena.

Statistical tests for swimming distances, head angle changes and individual tail parameters (Fig. 1D, Fig. 3F, G, Fig. 4C, G, Fig. 5B, Fig. S1D, Fig. S2F, Fig. S3D and Fig. S4A) used either 2-sample *t*-test or Tukey’s post-hoc test followed by one-way analysis of variance (ANOVA) in the Scipy package (https://scipy.org/). The statistical test for cortisol release against the control condition (Fig. 4B) used Dunnett’s multiple comparison test in the Scipy package. Statistical test for the change of the fraction of active neurons in the DRN (Fig. 6F) used Wilcoxon’s rank-sum test.

For independent component analysis (ICA) (Fig. 2D, Fig. 3H, Fig. 4E, Fig. 5C, F, Fig. S3E and Fig. S4C, D, E), we first identified ICA weights and normalization factors in the dataset of 14,694 swimming episodes from *N* = 22 fish for the small arena and *N* = 20 fish for the large arena (Fig. 1) by using FastICA function in scikit-learn package (https://scikit-learn.org). Then, we applied the same ICA weights and normalization factors to other datasets. Scatter density plots in this study (Fig. 2D, Fig. 3H, Fig. 4E, Fig. 5C, F, Fig. S3E, and Fig. S4C, D, E) were generated using the “*gaussian_kde”* function in Scipy package for dot coloring and *kdeplot* function in Seaborn package (https://seaborn.pydata.org/) for contour lines. Statistical tests for distributional differences of independent components (Fig. 3I, Fig. 4E, F, Fig. S2E, Fig. S4C, F and Fig. S5A, B) and zebrafish positions in the light-dark preference task (Fig. 4I) were performed using kernel density 2-sample test (kde.test) in R package through rpy2 Python-R bridge (https://pypi.org/project/rpy2/). We used this density-based test instead of Kolmogorov–Smirnov test because the former provides more conservative levels of significance for larger sample sizes [93].

The multi-drug comparison between psilocybin, ketamine and fluoxetine (Fig. 5G, H) was performed as follows. For distance-based comparison (Fig. 5G), we used the data presented in Fig. 3F, Fig. 4C, Fig. 5B and Fig. 5E. We calculated the stimulatory/suppressive effects based on changes of spontaneous swimming distances after drug application normalized by the mean of spontaneous swimming distance in the control group as below. The upper bars in the below equations represent averages between fish.

$$\frac{\,\left[{Spontaneous}\,{swim}\,{distance}\,{after}\,{drug}\,{application}\right]-\bar{[{Spontaneous}\,{swim}\,{distanceof}\,{control}\,{group}]}}{\bar{[{Spontaneous}\,{swim}\,{distance}\,{of}\,{control}\,{group}]}}$$

We calculated the anxiolytic effects based on how much the drug reversed the stress-induced changes in swimming distances as below.

$$\frac{[{Spontaneous}\,{swim}\,{distance}\,{after}\,{drug}\,{application}\,{and}\,{stress}\,{exposure}]-\bar{[{Spontaneous}\,{swim}\,{distance}\,{after}\,{stress}\,{exposure}]}}{\,\bar{[{Spontaneous}\,{swim}\,{distance}\,{after}\,{stress}\,{exposure}]}-\bar{[{Spontaneous}\,{swim}\,{distance}\,{of}\,{control}\,{group}]}}$$

We applied similar measures for the shifts in ICA components based on body kinematics (Fig. 5H). We calculated the stimulatory/suppressive effects based on changes in IC1 components during the spontaneous swimming of the drug-treated group compared to the mean of IC1 components of the control group. This was further normalized by the shifts in IC1 components by visual stimuli in the control group compared to the spontaneous swimming as below.

$$\frac{[{Spontaneous}\,{IC}1{compoments}\,{after}\,{drug}\,{application}]-\bar{[{Sopontaneous}\,{IC}1{components}\,{of}\,{control}\,{group}]}}{\bar{[{IC}1{components}\,{of}\,{control}\,{group}\,{during}\,{OMR}]}-\bar{[{Sopontaneous}\,{IC}1{components}\,{of}\,{control}\,{group}]}\,}$$

We calculated the anxiolytic effects based on how much the drug was able to reverse the stress-induced changes in IC2 components as below.

$$\frac{[{Spontaneous}\,{IC}2{after}\,{drug}\,{application}\,{and}\,{stress}\,{exposure}]-\bar{[{Spontaneous}\,{IC}2{after}\,{stress}\,{exposure}]}}{\bar{[{Spontaneous}\,{IC}2{after}\,{stress}\,{exposure}]}-\bar{[{Spontaneous}\,{IC}2{control}\,{group}]}}$$

### Neural activity imaging in the dorsal raphe nucleus

We used 5-day-old transgenic zebrafish that pan-neuronally express nuclear-localized, genetically-encoded calcium indicators [94] and co-express RFP in tph2+ neurons (Tg(HuC:H2B-GCaMP7f)^jf96^ and Tg(tph2:epNTR-TagRFP)^jf41Tg^, courtesy of Dr. Misha Ahrens). We used a custom dual-beam light-sheet microscope described in our previous work [79] to perform neural activity imaging. The zebrafish were immobilized and mounted to an imaging chamber as described previously [79]. Briefly, the fish larvae were immobilized by bath application of α-Bungarotoxin (B1601, Fisher Scientific, 0.5 mg/ml) dissolved in external solution (in mM: 134 NaCl, 2.9 KCl, 2.1 CaCl2, 1.2 MgCl2, 10 HEPES, 10 glucose; pH 7.8; 290 mOsm) for 45 s and embedded in 2% low-melting agarose on a custom-made pedestal inside a glass-walled chamber with a diffusive screen underneath the fish. The agarose around the head was removed with a microsurgical knife (#10318-14, Fine Science Tools) to minimize the scattering of the excitation laser. We acquired neural activity imaging data at the speed of one volume per second (45 planes per volume).

We performed electrical recordings of motor signals from the tail of the fish during the optomotor response task described in Fig. 6B. Electric signals from motoneuron axons in the tail were recorded using borosilicate pipettes (TW150-3, World Precision Instruments) pulled by a horizontal puller (P-1000, Sutter) and fire-polished by a microforge (MF-900, Narishige). The pipettes were filled with fish-rearing water and connected to the tail using minimal negative pressure. Swim signals were recorded using an amplifier (RHD2132 amplifier connected to RHD-2000 interface board, Intan Technologies). We used custom-written Python software (available upon request), a data interface card (NI PXIe-6341) and a terminal block (NI BNC-2110) for recording amplified signals at 6 kHz from the left and right channels. We used the same software and a compact projector (Optoma LV-130) to move visual stimuli projected to the bottom of the imaging chamber.

We processed imaging data on a Linux server in the High Performance Computing (HPC) division in the Weizmann Institute of Science. This server has two Xeon processors (Xeon Gold 6248, Intel), 384 GB RAM, 13-TB SSD array, and a GPU computing board (Tesla V100, nVidia). We performed data processing using custom Python scripts that execute the same algorithms as those established in our previous work [26] on a remote JupyterLab environment (https://jupyterlab.readthedocs.io/).

For data analysis, we first registered time-series images from the same Z-planes by using phase correlation algorithms on the above GPU. We then examined residual drifts in the lateral and axial directions and discarded data with excessive drifts (>5 μm) in either direction. We then identified individual neurons that express nuclear-localized GCaMP based on the average image by using an algorithm for detecting circular shapes in images. We then extracted fluorescent time series from the central part of identified neurons (49 pixels). We identified roughly 80,000 to 100,000 neurons across the brain. We calculated the baseline fluorescence trace for each extracted fluorescence trace by taking the rolling percentile of the bottom 30% with a window size of 2 min and then divided the original fluorescent time series by this baseline trace to obtain ΔF/F time series for each neuron.

To extract neural activity dynamics in the dorsal raphe nucleus, we registered our imaged brain into a reference brain (HuC:H2B-GCaMP) from the mapZebrain atlas (mapzebrain.org) using ANTs registration algorithm [95]. Three-dimensional coordinates of the above-segmented neurons are also registered to the reference brain space. Neurons in the dorsal raphe nucleus were further extracted using a “superior raphe” mask from the mapZebrain atlas. This anatomical mask covers the ventral and dorsal part of the raphe nucleus, and we used neurons that are dorsal than plane 159 for subsequent analysis in Fig. 6 (344 ± 61 neurons per fish).

We calculated the fraction of significantly activated neurons (Fig. 6F) as follows. For each neuron, we calculated the average ΔF/F values for the spontaneous period and the OMR period for each trial. We performed an independent *t*-test of these average ΔF/F values between two periods across trials, and neurons that showed *p*-values less than 0.05 were counted as significantly activated neurons. We calculated the fraction of such neurons for each period by dividing the number of significantly activated neurons by the total number of neurons in the above-described anatomical mask in each fish and performed a statistical test across different fish groups.

We analyzed how psilocybin affects latent neural population dynamics in the DRN by applying non-negative matrix factorization (NMF) to the trial-averaged activity of DRN neurons (Fig. 6G). We chose this non-negative method to orthogonalize complementary activity patterns of serotonergic populations and GABAergic populations in the DRN. We first calculated the trial average of ΔF/F time series (***S***_***n***_) from all neurons in the DRN and pooled their activity across all tested fish. We then applied NMF factorization in the direction of the neural population axis to obtain weights for individual neurons (***W***_***i,n***_) for the first two NMF components. We then calculated the normalized amplitudes of NMF components at each time point (***V***_***i,t***_) for the task for individual fish and a group of fish by the following equation

$${{{{{{\boldsymbol{V}}}}}}}_{{{{{{\boldsymbol{i}}}}}},{{{{{\boldsymbol{t}}}}}}}=\mathop{\sum}\limits_{{{{{{\boldsymbol{n}}}}}}}{{{{{{\boldsymbol{W}}}}}}}_{{{{{{\boldsymbol{i}}}}}}.{{{{{\boldsymbol{n}}}}}}}{{{{{{\boldsymbol{S}}}}}}}_{{{{{{\boldsymbol{n}}}}}},{{{{{\boldsymbol{t}}}}}}}$$

where ***i*** denotes the identification of NMF components, ***n*** denotes individual neurons, and ***t*** represents time points in a task presented in Fig. 6B. Amplitudes of resulting NMF components were normalized by its vector norm (***|V*** | ) for plotting traces in Fig. 6G.

### Homology analysis of serotonin receptors

The sequence homology analysis of serotonin receptors between zebrafish and human (Fig. 3B, Fig. S3A) was performed using Clustal Omega [96] algorithm and iTOL visualization tool [97] on the EMBL website. Uniplot IDs used for this analysis are as follows. Human serotonin receptors: hHTR1A (P08908), hHTR1B (P28222), hHTR1D (P28221), hHTR1E (P28566), hHTR1F (P30939), hHTR2A (P28223), hHTR2B (P41595), hHTR2C (P28335), hHTR3A (P46098), hHTR3B (O95264), hHTR3C (Q8WXA8), hHTR3D (Q70Z44), hHTR3E (A5X5Y0), hHTR4 (Q13639), hHTR5A (P47898), hHTR6 (P50406), hHTR7 (P34969). Zebrafish serotonin receptors: zHTR1aa (A0A8M1NIJ6), zHTR1ab (A0A8M1NRS3), zHTR1b (B3DK14), zHTR1d (A0A8M2B5P5), zHTR1e (A0A8M9P2V8), zHTR1fa (A0A8M6Z176), zHTR1fb (A0A8M2B6K6), zHTR2aa (A0A8N7TD42), zHTR2ab (A0A8M3B093), zHTR2b (Q0GH74), zHTR2cl1 (A0A8M6Z717), zHTR2cl2 (A0A8M1PZA4), zHTR3a (A0A8M9PD95), zHTR3b (A0A8M9PJB8), zHTR4 (A0A8M9QPE9), zHTR5aa (A0A8M1NJ85), zHTR5ab (Q7ZZ32), zHTR6 (A0A8M3ANX4), zHTR7a (A0A8N7T7N6), zHTR7b(A0A8M9QGY4), zHTR7c (A0A8M1RQY0).

### Histology

zHTR2cl1 expression map (Fig. 3C) was constructed using RNA fluorescence in situ hybridization (RNA-FISH) with hybridization chain reaction (HCR) method [98]. HCR probe for zHTR2cl1, amplifiers and buffers were purchased from Molecular Instruments. The staining was performed according to an HCR protocol provided by Dr. Inbal Shainer (Max Planck Institute for Biological Intelligence, Germany) [99] by using a B3 amplifier with Alexa Fluor 546. We used 5-day-old transgenic zebrafish that pan-neuronally express nuclear-localized, genetically-encoded calcium indicator (Tg(HuC:H2B-GCaMP7f)) for the staining to register the volumetric image to a reference brain based on GCaMP expression.

Labeled fish were imaged in our custom light-sheet microscope [79]. Prior to the imaging, fish were embedded in 2% low-melting agarose in fish water on a custom-made pedestal inside a glass-walled chamber. The agarose around the head was removed with a microsurgical knife (#10318-14, Fine Science Tools) to minimize the scattering of the excitation laser. For each fish, we acquired two images: Tg(HuC:H2B-GCaMP7f) channel and the zHTR2cl1 HCR staining image.

Image stacks were processed using custom Python scripts on a remote JupyterLab environment in the above-mentioned server. We created an average image for each fish. We then identified individual neurons that express nuclear-localized GCaMP based on the average image by using an algorithm for detecting circular shapes in images. Then, we registered the GCaMP image channel of each fish to Tg(elavl3:H2b-GCaMP6s) reference image from the mapZebrain database using Advanced Normalization Tools (ANTs) [95] and applied the same registration to the HCR in situ image. For each fish, we analyzed the HCR signal in the coordinates of the recognized neurons after subtracting the local background and created a binarized image of the signal. Then, we overlaid a spherical Gaussian filter of the positive neurons of each fish, normalized by the number of cells, to create a generalized 3D image of expressing regions.

The anatomical image of serotonergic and GABAergic neurons in the DRN (Fig. 6D) was obtained by conducting whole-mount immunohistochemistry of a transgenic zebrafish that expresses RFP in *gad1b*+ neurons Tg(gad1b:loxP-RFP-loxP-GFP)^nns26Tg^ [100] using anti-5-HT rabbit polyclonal antibody (S5545, Sigma-Aldrich) as described in our previous work [26].

### Enzyme-linked immunosorbent assay (ELISA)

For ELISA preparation, we utilized the stress paradigm described in Fig. 4A and then extracted cortisol from fish. We waited for 5 min after the end of stress exposure and then beheaded the larval fish with a microsurgical knife (#10318-14, Fine Science Tools) to stop the interaction between the brain and adrenal glands. 4–8 fish were placed in each tube to serve as a single sample. We prepared 3-4 samples per condition for each batch. The fish were then stored at −80^o^C. On the day of the ELISA assay, each tube was homogenized with magnet beads. Cortisol was extracted by adding 5 ml of ether into each tube. The ether was separated from the water and vaporized in a 60 ^o^C bath. We then re-suspended the cortisol in 60 μl of steroid assay buffer.

We coated polystyrene ELISA microtiter plates (Nunc-Immuno^TM^ Plates, Nunc) with 100 μl/well of anti-cortisol antibody diluted to 10 µg/ml in carbonate buffer (50 mM sodium carbonate, pH 9.6), and incubated for 2 h at 37 ^o^C without shaking. Two control wells for non-specific binding were not coated. After 2 h, plates were washed (3 × 5 min) with 200 μl/well of Wash buffer (20 ml Solution C, 0.5 ml Tween 20, filled to 1 L with distilled water)

The standard curve (Fig. S3F) was prepared by creating a series of 10 tertiary (1:3) dilutions of standard cortisol diluted in steroid assay buffer, with an initial conc. of 200 ng/ml and a final volume of 400 µl in each tube. Steroid assay buffer (SAB) consists of 100 ml Solution C [370 mg EDTA, 1 g BSA, 150 µl Sodium azide], and filled up to 1 L with distilled water with a pH of 7.4. 125 µl of each standard were mixed with 125 µl/well of HRP-conjugated cortisol diluted 1:1280 in SAB. 100 µl of the solution was then transferred to the ELISA plate and divided into duplicate wells.

Samples were prepared by adding 75 µl of SAB and 125 µl of HRP-conjugated cortisol diluted 1:1280 in SAB to 50 µl of the sample, which was mixed well by pipetting. Each sample was dispensed into the wells (100 μl/well) of the coated microtiter plates and incubated for 1 h at room temperature in the dark without shaking. Following incubation, the plates were washed (3 × 5 min) with a Wash buffer. The presence of enzyme complexes was visualized by the addition of 100 μl/well of 3,3′,5,5′-tetramethylbenzidine (TMB) peroxidase substrate (KPL, Zotal, Israel). The reaction was carried out in complete darkness at RT and was stopped after 45 min with TMB stop solution (100 μl/well). Absorbance was read at 450 using a Spectra II ELISA reader (SLT, Salzburg, Austria).

We experienced large variations in measured cortisol levels across fish and batches. Therefore, batches that have large variance within themselves (coefficient of variation>1) were excluded from the analysis for the concern of suboptimal cortisol extractions. We normalized the fluctuation of cortisol values across batches with the average of each batch (0.15 to 3.1 ng per larvae) for the statistical analysis in Fig. 4B. Cortisol measurements that went below the range of standard curve were set to 0 for three samples in the control condition.

### Supplementary information

Supplementary Figure

## Data Availability

Body kinematics data of free-swimming zebrafish under various drug treatment conditions are available upon request.

## Code availability

Custom Python scripts used for data processing, data visualizations and statistical analyses in this study are available upon request.
