# Supplementary material for: High-resolution tracking of unconfined zebrafish behavior reveals stimulatory and anxiolytic effects of psilocybin
Source: Mol Psychiatry. 2024 Jan 17;29(4):1046–62. doi: 10.1038/s41380-023-02391-7 (PMC11176078; doi:10.1038/s41380-023-02391-7)
Supplement: Supplementary file 1 — Supplementary Figure [file 41380_2023_2391_MOESM1_ESM.pdf]

Fig. S1: Experimental setup

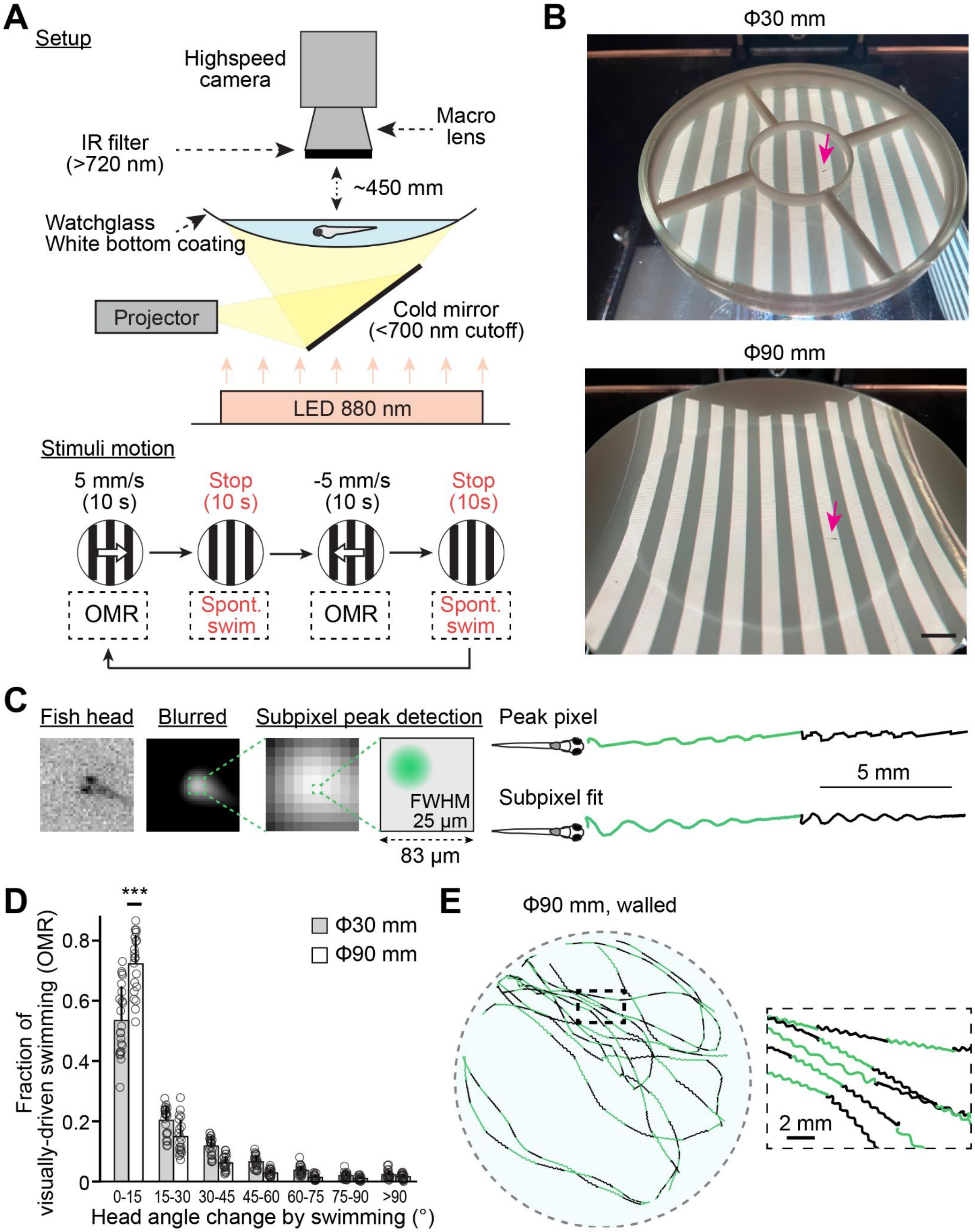

Fig. S1: Experimental setup

(A) *Top*, our experimental setup for zebrafish tracking in a large environment. Fish behavior is measured in a concave chemical watch glass (for the large environment) or a plastic insert (for the small environment), of which the bottom surface is coated with a white spray. A projector showed visual stimuli in the bottom of the dish at visible wavelength. An infrared illumination was placed under the dish, and a high-speed camera with a macro lens acquired images through an infrared filter. *Bottom*, cycles of visual stimulus motion during the experiment. We define optomotor response (OMR) as swim events observed during visual stimulus motion (black) and spontaneous exploration as those observed when the visual stimulus is stopped (red). (**B**) Photos of actual experiments in the small (30 mm) and the large (90 mm) dish, with arrows to help locate the fish. **(C)** Subpixel peak detection significantly improves the accuracy of head centroid trajectories. **(D)** Increased straight swimming during optomotor response in the large arena compared to the small arena. ***, $p=1.8*10^{-6}$ from 2-sample t-test. Error bars represent standard deviations across tested fish. **(E)** Fish's swim patterns in a large environment (90 mm) with a flat floor and a boundary wall. *Left,* head centroid trajectories of a single fish. *Right,* expanded head centroid trajectories from the outlined central parts of the large arena on the left.

Fig. S2: Recognition of tail motions and its accuracy validation

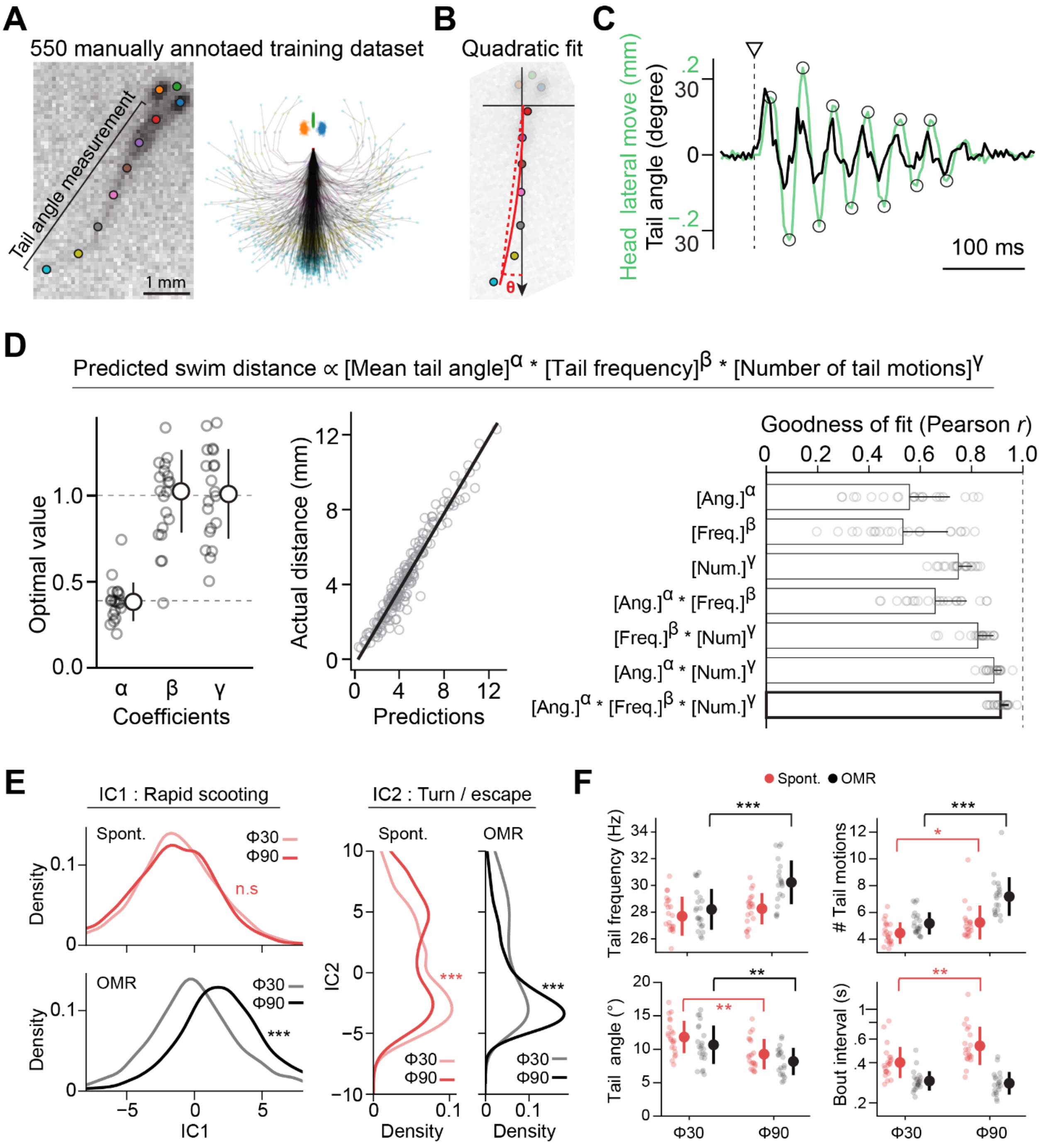

Fig. S2: Recognition of tail motions and its accuracy validation

(A) *Left*, annotation of 10 points across the body. *Right*, distribution of body parts in 550 manually annotated training datasets. Manual annotations were made using fish images from various orientations, and we aligned them for the visualization of this panel. Training images were selected to balance various tail angles on the left and right sides. **(B)** Tail angle θ was quantified by fitting a quadratic function to annotated points along the tail. **(C)** Overlay of tail motions and head centroid motions during a representative swim episode. Peaks of head centroid motions were detected (circles) and used as a reference to quantify tail motions. **(D)** Validating accuracies of tail angle quantification by predicting swimming distance based on tail motion parameters. *Left*, parameters of a multiplicative prediction model (left) were optimized by using an optimizer. *Center*, the resulting model shows high correlations to swimming distance. *Right*, quantification of the prediction accuracy tested in a large environment. The full model (bottom) has an accuracy of Pearson correlation coefficient $r = 0.89 \pm 0.036$ across 20 fish. Error bars represent standard deviations. **(E)** Statistical analyses of IC1 and IC2 were performed using the same set of fish in Fig. 1 and Fig. 2 by using kernel density 2-sample test (see Methods). We included 2,635 (small arena) and 5,233 (large arena) swim episodes for the statistics of spontaneous exploration and 1,466 (small arena) and 5,360 (large arena) swim episodes for the statistics of optomotor response. IC1: n.s. (not significant), p=0.14; ***, p=6.0*10^{-49}. IC2: ***, p=1.4*10^{-12} and 1.1*10^{-4} for spontaneous exploration and optomotor response, respectively. **(F)** Analyses of individual swim parameters. The large arena facilitated significantly higher frequencies, more numbers of tail motions and smaller tail angles during optomotor response, while it elongated bout intervals during spontaneous exploration. P values are from a 2-sample t-test between N=22 and N=20 fish for the small and large dishes, respectively. ***, p=2.3*10^{-4} (frequency); *, p=0.021, ***, p=2.8*10^{-6} (motions); ** for spontaneous exploration; **, p=1.4*10^{-3}, **, p=3.2*10^{-3} (angle); **, p=4.1*10^{-3} (interval) for optomotor response. Error bars represent standard deviations across tested fish.

Fig. S3: Behavioral effects of fluoxetine and fluvoxamine treatment

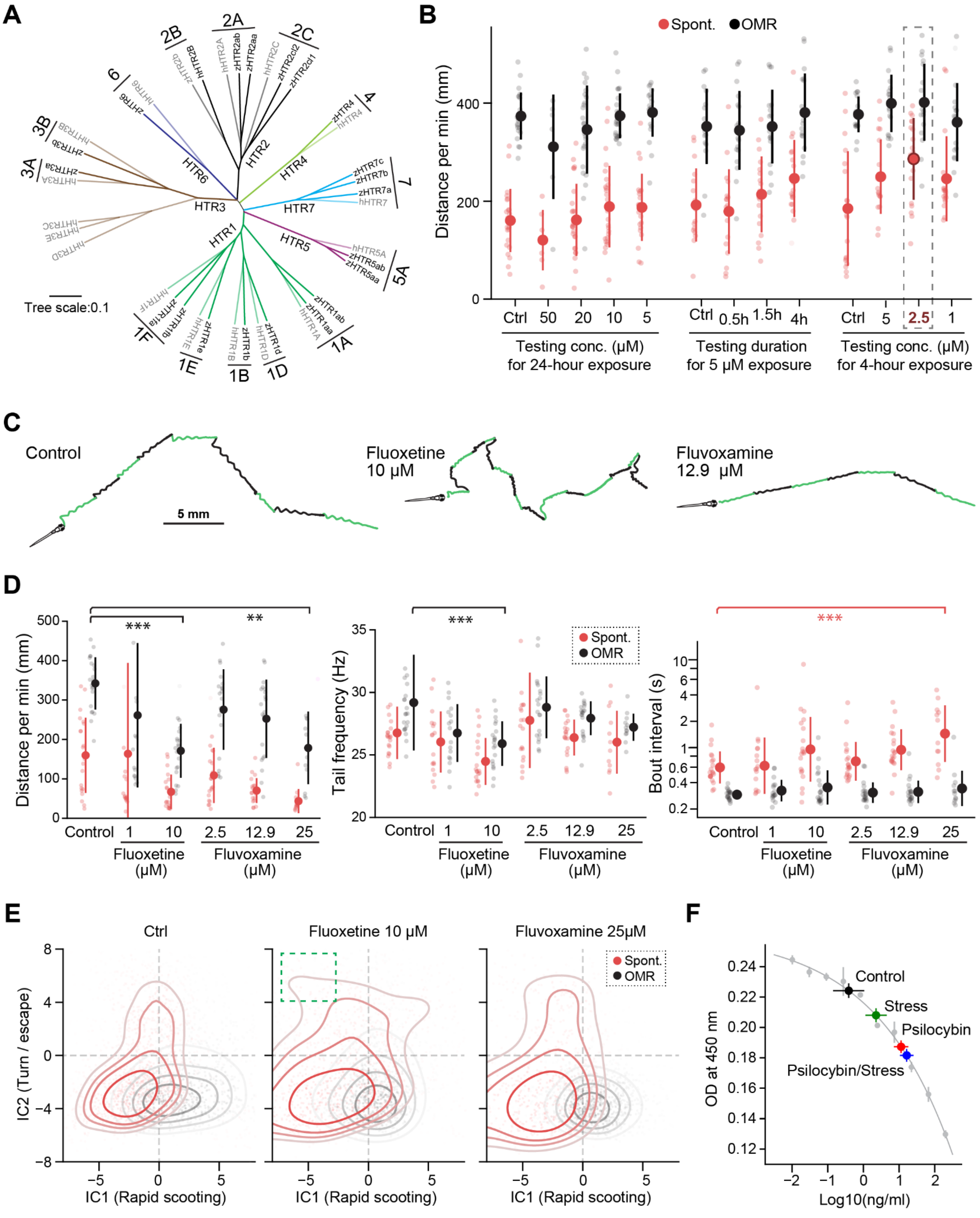

Fig. S3: Behavioral effects of fluoxetine and fluvoxamine treatment

(A) Unbiased homology analyses of protein sequences of all serotonin receptors revealed conserved major types and subtypes between zebrafish and humans. **(B)** The effect of psilocybin treatment on swimming distances during spontaneous exploration (red) and optomotor response (black). We tested various durations and concentrations (conc.) of psilocybin exposure. Data from the same set of experimental batches are clustered together with their individual control data. Numbers of fish (left to right): N=22 (Ctrl), 6 (50 μM), 25 (20 μM), 26 (10 μM), 21 (5 μM), 17 (Ctrl), 18 (0.5h), 18 (1.5h), 18 (4h), 18 (Ctrl), 18 (5 μM), 18 (2.5 μM) and 18 (1 μM). The data on the right (4-hour exposure) is the same as those shown in Fig. 3F. The condition in the dashed box (2.5 μM, 4 h) had the strongest impact on spontaneous exploration and was used for experiments in Fig. 3 and Fig. 4. **(C)** Swim trajectories of control, fluoxetine-treated, and fluvoxamine-treated fish during optomotor response. **(D)** Swimming distance per minute (left), tail frequency (center) and average tail angle (right) of control (N=21), fluoxetine-treated (N=17 for 1 μM, N=20 for 10 μM) and fluvoxamine-treated fish (N=20 for 2.5 μM, N=20 for 12.9 μM, N=12 for 25 μM). P values are from Tukey's post-hoc test after one-way ANOVA analysis. ***, $p=3.8*10^{-5}$; **, $p=1.1*10^{-3}$ (distance per minute during optomotor resopnse); ***, $p=6.4 * 10^{-4}$ (tail frequency during optomotor response); ***, $p=2.5*10^{-4}$ (bout intervals during spontaneous exploration). Error bars represent standard deviations. **(E)** Independent component analysis (ICA) of swim patterns after the sham treatment (left), fluoxetine 10 μM treatment (center) and fluoxetine 25 μM treatment (right). Data from 32, 20 and 12 fish were plotted, respectively. The loci of odd swim patterns in the ICA space after fluoxetine treatment are indicated with a dashed green box. **(F)** Example of a standard curve and sample measurement in the ELISA assay for quantifying cortisol from a single batch. Cortisol standards ranging from 200 ng/ml to 0.01 ng/ml (gray dots) were used for calibrating a standard curve for quantifying cortisol in larval zebrafish under various conditions. Error bars represent standard deviation.

Fig. S4: Behavioral changes after hypertonic stress exposure

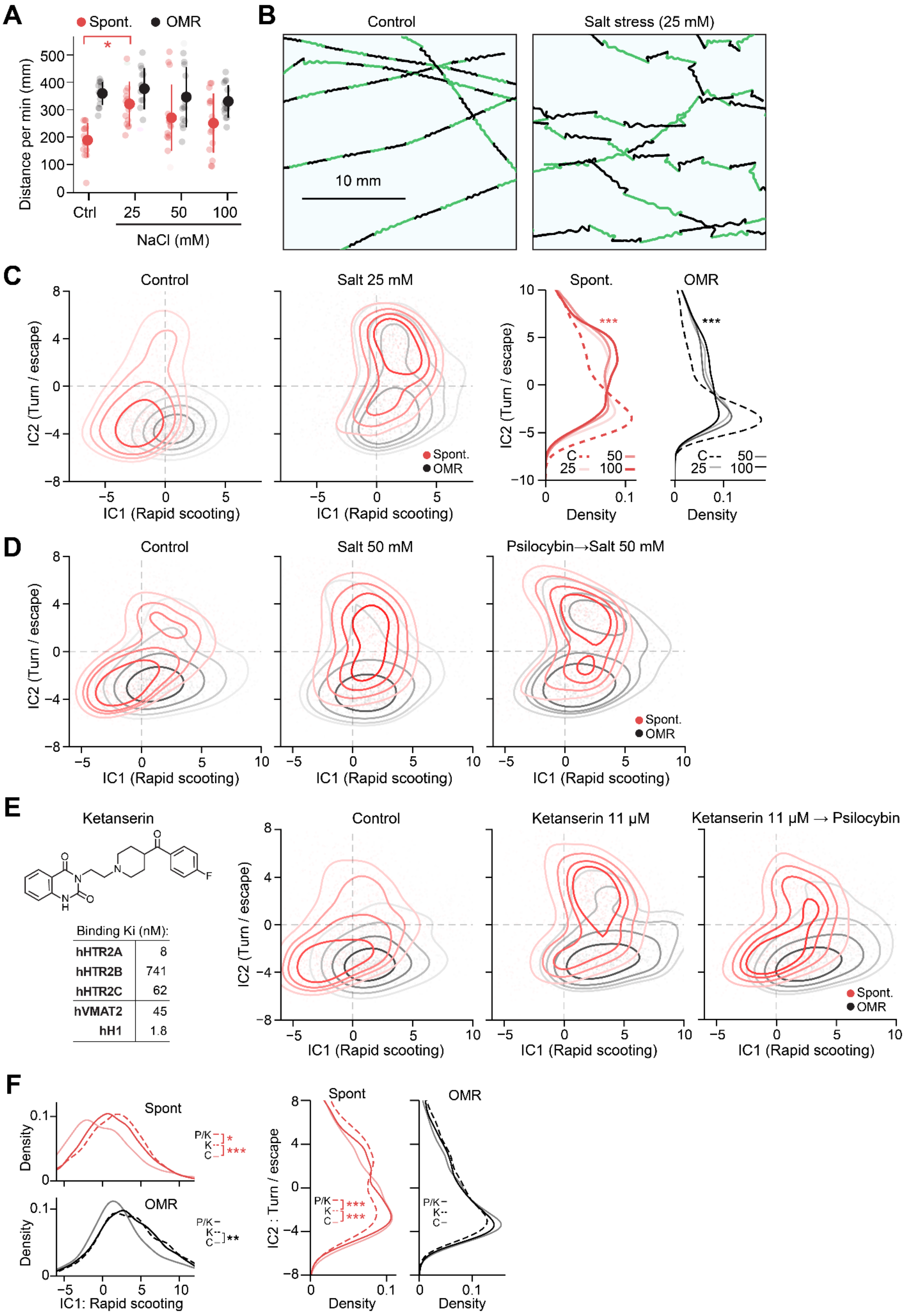

Fig. S4: Behavioral changes after hypertonic stress exposure

(A) Swimming distance during spontaneous exploration and optomotor response after exposure to varying concentrations (25 mM, 50 mM and 100 mM) of sodium chloride in the water as hypertonic stress. N=14 fish for all conditions. We used Tukey's post-hoc test following one-way ANOVA analysis of spontaneous exploration among different conditions for statistics. *, p=0.049. Error bars represent standard deviations. **(B)** Head centroid trajectories around the central part of the large arena after sham treatment (left) and hypertonic stress exposure (right) during optomotor response. **(C)** *Left*, independent component analysis (ICA) revealed the shift of swim patterns toward turn/escape behavior (IC2) after hypertonic stress exposure. The same number (1200) of randomly selected swim events were plotted for each condition. *Right,* statistical analyses of the occurrences of turning/escape behaviors based on the IC2 component. We used kernel density 2-sample test (see Methods) for statistics. We included 5,227 (control), 6,259 (25 mM), 5,514 (50 mM) and 5,046 (100 mM) swim episodes for the statistics of spontaneous exploration, and 4,730 (control), 3,322 (25 mM), 3,370 (50 mM) and 3,267 (100 mM) swim episodes for the statistics of optomotor response. ***, $p=4.2*10^{-28}$ (control vs 100 mM for spontaneous exploration); ***, $p=4.9*10^{-5}$ (control vs 100 mM for optomotor response). **(D)** Independent component analysis (ICA) of swim patterns after the sham treatment (left), hypertonic stress exposure (center) and psilocybin pretreatment and stress exposure (right). Data from 11 fish for each condition. **(E)** *Left*, the structure of ketanserin and its affinities to binding targets. Affinity values for HTR2 receptors are from ref[72], and those for vesicular monoamine transporter 2 (VMAT2) and type 1 histamine receptor (HT1) are from ref[73] and ref[74], respectively. *Right*, independent component analysis (ICA) of swim patterns after the sham treatment (C), ketanserin exposure (K) and psilocybin/ketanserin exposure (P/K). Data from 14, 15 and 15 fish for each condition, respectively. **(F)** Statistical analyses of the behavioral shifts along the IC1 and IC2 axes during ketanserin/psilocybin treatments presented in (E). We included 3,644 (C), 4,315 (K) and 4,106 (P/K) swim episodes for the statistics of spontaneous exploration and 3,108 (C), 3,101 (K) and 3,134 (P/K) swim episodes for the statistics of optomotor response. IC1 statistics are as follows: ***, $p=1.7*10^{-72}$ (C vs K); *, p=0.015 (K vs. P/K) during spontaneous exploration. **, $p=7.4*10^{-3}$ (C vs K) during optomotor response. IC2 statistics are as follows: ***, $p=6.9*10^{-8}$ (C vs K); ***, $p=4.7*10^{-5}$(K vs. P/K) during spontaneous exploration.

Fig. S5: Statistics of ketamine- and fluoxetine-treated fish

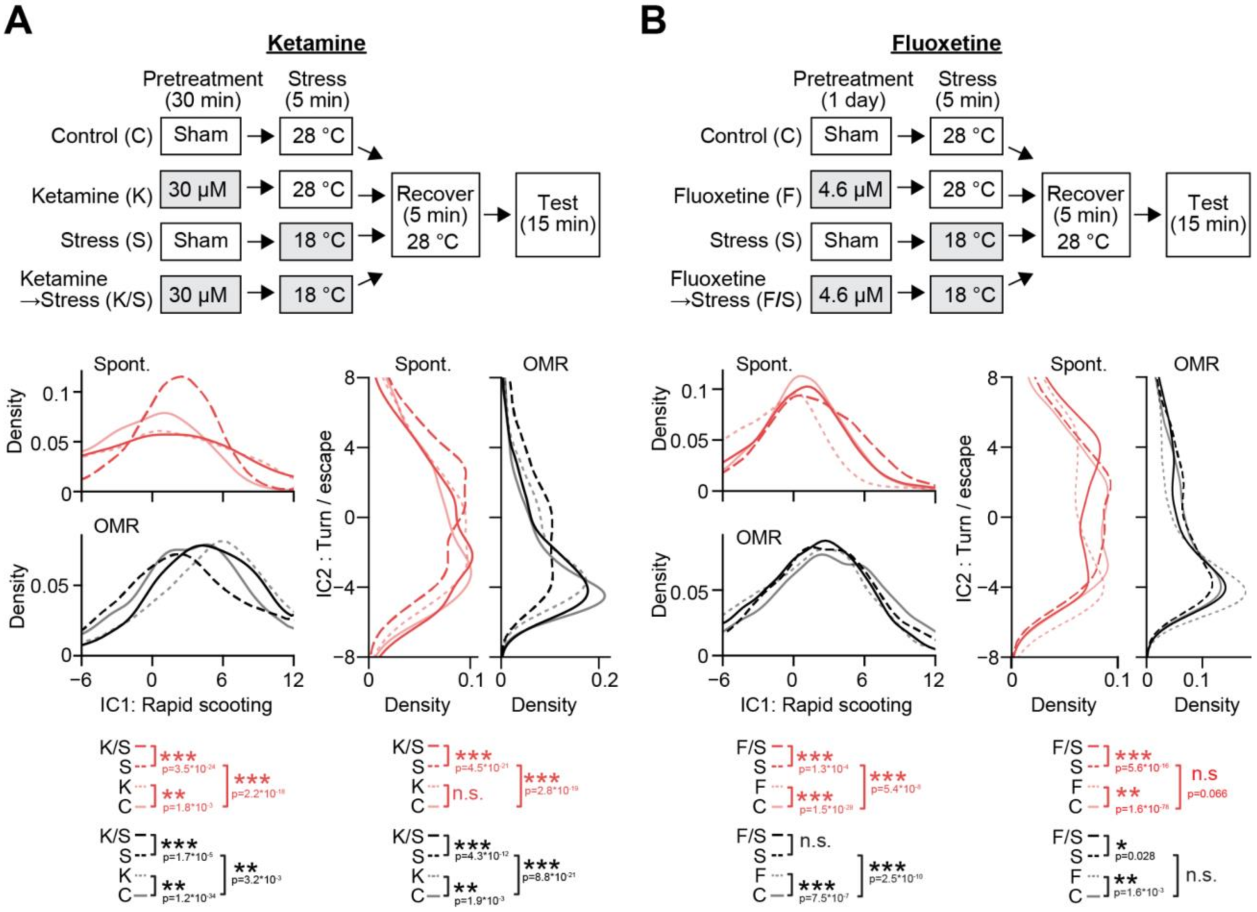

(A) *Top,* behavioral paradigm for ketamine and cold shock treatment**.** *Bottom,* statistical analyses of the IC1 and IC2 components were performed using the same set of fish in Fig. 5C by using kernel density 2-sample test (see Methods). C, Control; K, ketamine-treated; S, stressed; K/S ketamine-treated/stressed. We included 1297, 670, 2869, and 723 (for C, K, S and K/S) swim episodes for the statistics of spontaneous exploration and 2048, 1353, 1919 and 966 (for C, K, S and K/S) swim episodes for the statistics of optomotor response. Statistical results and p-values are shown below the graph. **(B)** *Top,* behavioral paradigm for fluoxetine and cold shock treatment**.** *Bottom,* statistical analyses of the IC1 and IC2 components were performed using the same set of fish in Fig. 5E by using kernel density 2-sample test (see Methods). C, Control; F, fluoxetine-treated; S, stressed; F/S fluoxetine-treated/stressed. We included 4845, 4227, 7220 and 6319 (for C, F, S and F/S) swim episodes for the statistics of spontaneous exploration and 4371, 5291, 4282 and 5034 (for C, F, S and F/S) swim episodes for the statistics of optomotor response. Statistical results and p-values are shown below the graph.
